# A ZDHHC3–USP5–PTRF axis links palmitoylation to ferroptosis-associated phenotypes in melanoma

**DOI:** 10.1038/s41419-026-09053-w

**Published:** 2026-07-02

**Authors:** Songyun Zhao, Yuankun Liu, Dan Wu, Chenfeng Ma, Peng Luo, Pengpeng Zhang, Yucang He, Xiaoqing Liang, Chao Cheng, Jiaheng Xie, Liqun Li

**Affiliations:** 1 Department of Plastic Surgery, The First Affiliated Hospital of Wenzhou Medical University, Wenzhou, China; 2 Department of Neurosurgery, The Affiliated Wuxi People’s Hospital of Nanjing Medical University, Wuxi, China; 3 Department of Dermatology, Huashan Hospital of Fudan University, Shanghai, China; 4 Department of Neurosurgery, The First Affiliated Hospital of Nanjing Medical University, Nanjing, China; 5 Department of Oncology, Zhujiang Hospital of Southern Medical University, Guangzhou, China; 6 Department of Lung Cancer Surgery, Tianjin Medical University Cancer Institute and Hospital, Tianjin, China; 7 Department of Oncology, Beijing Shijitan Hospital, Capital Medical University, Beijing, China; 8 Department of Plastic Surgery, Xiangya Hospital Central South University, Changsha, China

**Keywords:** Oncogenes, Cell death

## Abstract

Melanoma is a highly aggressive malignancy with poor prognosis and therapy resistance. Although deubiquitinating enzymes (DUBs) regulate protein stability and cancer progression, their modulation in melanoma remains unclear. Here, we characterized ubiquitin‑specific peptidase 5 (USP5) using proteomic, biochemical, and functional approaches. USP5 was upregulated in melanoma and promoted proliferation, migration, and invasion. Mechanistically, USP5 directly interacted with and deubiquitinated PTRF, preferentially removing K48‑linked polyubiquitin chains to reduce proteasomal degradation. PTRF represents a functionally relevant substrate, though additional targets may exist. USP5 stability itself depended on S‑palmitoylation mediated by the palmitoyltransferase ZDHHC3, which suppressed USP5 ubiquitination and turnover. Silencing ZDHHC3 destabilized USP5, reduced PTRF levels, and impaired tumor growth. Perturbing the ZDHHC3–USP5–PTRF axis modulated ferroptosis‑associated phenotypes in vitro and affected xenograft tumor growth, accompanied by altered oxidative damage markers indicative of redox and lipid peroxidation changes, without directly establishing ferroptosis dependency. Our findings identify ZDHHC3‑mediated palmitoylation as a key determinant of USP5 stability and reveal a palmitoylation–deubiquitination cascade with PTRF as a downstream effector, linking this pathway to ferroptosis‑related cellular phenotypes. Further in vivo studies are needed to clarify mechanistic specificity and ferroptosis dependency.

## Introduction

Skin cutaneous melanoma (SKCM) is a highly malignant tumor originating from melanocytes, characterized by pronounced invasiveness and metastatic potential. In recent years, its global incidence has continued to rise, making it a major disease that poses a serious threat to public health [1]. Despite advances in immunotherapy, targeted therapy, radiotherapy, and chemotherapy, melanoma still exhibits strong metastatic tendencies and drug resistance, leading to poor overall prognosis in patients [2]. At the molecular level, melanoma development is closely associated with abnormalities in multiple key signaling pathways and regulatory networks. Post-translational modifications (PTMs) of proteins regulate cell proliferation, metabolism, and immune responses by altering protein conformation, localization, and activity, thereby playing an irreplaceable role in tumorigenesis and progression [3].

Among the various PTMs, the ubiquitin–proteasome system (UPS) serves as the central mechanism maintaining cellular protein homeostasis. It not only mediates the degradation of abnormal proteins but also broadly participates in signal transduction and stress regulation [4]. Substantial evidence indicates that the UPS plays a pivotal role in melanoma oncogenesis by degrading tumor suppressor proteins, sustaining oncogene activity, and facilitating immune evasion [5]. As key components of the UPS, deubiquitinating enzymes (DUBs) can reverse ubiquitination, thereby stabilizing or activating specific substrates [6]. Dysregulated activation of DUBs is not only associated with tumor initiation and progression but also contributes to immunosuppression and resistance to targeted therapies, making them promising therapeutic targets [7–9].

Ubiquitin-specific peptidase 5 (USP5), also known as isopeptidase T (ISOT), is an important member of the USP family and has been reported to be abnormally overexpressed in several malignancies, including breast, lung, colorectal, and liver cancers [10, 11]. For example, USP5 promotes non-small cell lung cancer progression by suppressing p53-dependent senescence via the USP5–BECLIN1 axis [12]; in breast cancer, DEPDC1B-dependent USP5 stabilizes β-catenin and activates Wnt/β-catenin signaling to promote metastasis [13]. Our previous study also revealed that USP5 enhances immune evasion in melanoma by deubiquitinating and stabilizing PD-L1, providing new mechanistic insights into immunotherapy resistance [14]. Collectively, these findings indicate that USP5 possesses strong oncogenic potential in melanoma; however, its upstream regulation and downstream effects remain incompletely understood.

S-palmitoylation, one of the most common and reversible lipid modifications, involves the covalent attachment of palmitic acid to cysteine residues of proteins via thioester bonds [15]. This modification affects protein membrane localization, stability, and subcellular distribution, playing crucial roles in cell growth, survival, and drug response. Increasing evidence suggests that dysregulated palmitoylation is closely linked to the initiation and progression of various cancers [16]. Twenty-three palmitoyltransferases containing a conserved ZDHHC motif catalyze this modification—for instance, ZDHHC8 mediates the palmitoylation of SLC7A11 at Cys327, thereby inhibiting its ubiquitination and degradation [17]. Given its druggable nature in tumors, palmitoylation has emerged as a potential entry point for precision therapy [18, 19].

In this study, we identify a ZDHHC3–USP5–PTRF regulatory cascade in melanoma cells. We show that ZDHHC3-dependent S-palmitoylation is associated with USP5 protein stability, and that USP5 interacts with PTRF and counteracts its ubiquitination to maintain PTRF abundance. While PTRF emerges as a relevant downstream substrate, it is unlikely to represent the sole effector of USP5 function. Given prior evidence linking PTRF to cellular redox regulation, the USP5–PTRF axis may contribute to ferroptosis-associated phenotypes. However, because DUB activity and ferroptosis responses are context-dependent, the mechanistic and in vivo relevance of this pathway requires further validation.

## Materials and methods

### Single-cell sequencing analysis

Public single-cell transcriptomic data were obtained from the GEO dataset GSE215120 (including 6 acral melanoma [AM] and 3 cutaneous melanoma [CM] cases) [20]. h5-format data were imported with the Read10X_h5 function and processed in Seurat (v4.2.0). Quality-control filters were: genes detected per cell 200–7,000; mitochondrial gene fraction <20%; hemoglobin gene fraction <10%. Batch effects were corrected using canonical correlation analysis (CCA), and doublets were removed with DoubletFinder [21]. Data were normalized with NormalizeData, highly variable genes were identified with FindVariableFeatures, and dimensionality reduction was performed with ScaleData (post–batch correction), PCA, and t-SNE/UMAP. Cell clusters were annotated using known marker genes. Differential expression was performed with FindMarkers; pathway enrichment used GSVA and GSEA against Hallmark gene sets. Spatial transcriptomics (ST) melanoma data were downloaded from 10x Genomics. ST data were analyzed in Seurat, including UMI count normalization, scaling, and selection of the most variable features via SCTransform, followed by RunPCA for dimensionality reduction and unsupervised clustering. SpaCET was used to infer cancer cell abundance by integrating a gene-pattern dictionary across common malignancies; a constrained linear regression model calibrated local tissue density, and stromal and immune lineage scores were derived based on a comprehensive non-malignant cell atlas [22].

### Cell culture

Human melanoma cell lines A375 and SK-MEL-28, embryonic kidney cells HEK-293T, and immortalized human melanocytes PIG1 were purchased from the National Cell Resource Center. All cells were cultured at 37 °C with 5% CO₂ under saturated humidity. A375, SK-MEL-28, and HEK-293T were maintained in high-glucose Dulbecco’s Modified Eagle Medium (DMEM, Gibco) supplemented with 10% fetal bovine serum (FBS, Gibco), 2 mM L-glutamine, 100 U/mL penicillin, and 100 µg/mL streptomycin. PIG1 cells were cultured in Ham’s F-10 medium (Gibco) with 10% FBS, 2 mM L-glutamine, and human melanocyte growth supplements as recommended by the supplier. Cells at 70–90% confluence were passaged with 0.05% trypsin-EDTA at a routine split ratio of 1:3–1:6. Cells used for experiments were within 10 passages post-thaw. All cell lines tested negative for mycoplasma. For cryopreservation, complete medium containing 10% DMSO was used; for recovery, cells were promptly transferred into prewarmed culture medium and expanded.

### Transfection and gene silencing/overexpression

Lentivirus was produced using a transient transfection system in HEK-293T cells. Lipofectamine 3000 (Thermo Fisher Scientific) was used to co-transfect shRNA vectors targeting USP5 (sh-USP5) or ZDHHC3 (sh-ZDHHC3) with packaging plasmids psPAX2 and pMD2.G at recommended ratios. Viral supernatants were collected at 48 h and 72 h post-transfection, filtered through 0.45 µm membranes, and used to infect A375 and SK-MEL-28 cells in the presence of 8 µg/mL polybrene (Sigma). Medium was replaced after 24 h, and stable knockdown lines were selected with 2 µg/mL puromycin for 5–7 days. For transient overexpression, plasmids used as required included Flag-USP5-WT, the catalytically inactive mutant Flag-USP5-C335A, HA-Ub (WT, K6, K11, K27, K48, or K63), Myc-ZDHHC3-WT, and the catalytically inactive Myc-ZDHHC3-CS. Cells were harvested 36–48 h post-transfection for protein/RNA extraction and functional assays. To block proteasomal degradation in ubiquitination-related experiments, 10 µM MG132 (Selleck) was added 6 h prior to lysis. Knockdown and overexpression efficiencies were verified by Western blot.

### Quantitative real-time PCR (qPCR)

Total RNA was extracted with TRIzol (Invitrogen), and concentration/purity were measured by NanoDrop. One microgram of RNA was reverse-transcribed into cDNA using PrimeScript RT or SuperScript III (Takara/Thermo Fisher Scientific), with genomic DNA removal as needed. qPCR was performed on an ABI 7500 system in 20 µL reactions (10 µL SYBR Green mix, 2 µL cDNA, 0.4 µM each primer, nuclease-free water to volume). Cycling: 95 °C for 3 min; then 40 cycles of 95 °C for 10 s and 60 °C for 30 s; followed by melt-curve analysis to confirm specificity. Primer sequences are listed in Supplementary Table 1. Primer efficiencies were 90–110% with single-peak melt curves. Each sample was run in technical triplicate with at least three independent biological replicates. Relative expression was calculated by the 2^(-ΔΔCt) method with β-Actin as the internal control. Data are presented as mean ± SD; statistical methods are detailed in the Statistics section.

### Colony formation and transwell assays

For colony formation, 500–1000 cells per well were seeded in 6-well plates and cultured for 10–14 days, then fixed with 4% paraformaldehyde and stained with crystal violet. Colonies with >50 cells were counted. Migration/invasion assays used Transwell chambers with 8 μm pores; invasion chambers were pre-coated with Matrigel. Cells were pretreated with mitomycin C (10 μg/mL) for 2 h, washed, and then suspended in serum-free medium before seeding into the upper chamber, with medium containing 10% FBS in the lower chamber as a chemoattractant. After 24–48 h, non-migrated cells were removed; migrated/invaded cells were fixed, stained with crystal violet, and counted microscopically. Detailed procedures followed our previously published protocol [23].

### Affinity purification–LC–MS/MS for USP5 substrate identification

To identify USP5-interacting substrates, Flag-USP5 or empty-vector control was stably or transiently expressed in A375 and SK-MEL-28 cells. Cells were lysed on ice in IP buffer (50 mM Tris-HCl pH 7.5, 150 mM NaCl, 0.5% NP-40, 1 mM EDTA, protease inhibitors, 20 mM N-ethylmaleimide), and debris was cleared by centrifugation at 4 °C. Clarified lysates were incubated with anti-Flag magnetic beads (Sigma or Thermo Fisher Scientific) for 2 h, followed by five washes at 4 °C, including one with high-salt buffer (300 mM NaCl) to reduce nonspecific binding. Bound proteins were reduced with DTT, alkylated with iodoacetamide, and on-bead digested overnight at 37 °C with sequencing-grade trypsin (Promega). Peptides were acidified, desalted on C18 SPE, lyophilized, and reconstituted in 0.1% formic acid. After nano-LC separation on a C18 column, peptides were analyzed online on a high-resolution Orbitrap mass spectrometer (Thermo Fisher Scientific) with full MS1 and data-dependent MS/MS acquisition. Raw data were processed in MaxQuant against the UniProt human database with 1% FDR at PSM and protein levels, enabling label-free quantification (LFQ). Common contaminants, reverse hits, and single-peptide identifications were removed. Proteins significantly enriched in the Flag-USP5 group versus empty vector and IgG controls (log₂ LFQ fold change ≥1 and adjusted *P* < 0.05) were considered candidate interactors for validation.

### Western blotting

Cells were washed with ice-cold PBS and lysed in RIPA buffer containing protease and phosphatase inhibitors (50 mM Tris-HCl pH 7.4, 150 mM NaCl, 1% NP-40, 0.5% sodium deoxycholate, 0.1% SDS). Lysates were cleared at 14,000 × *g* for 10 min at 4 °C. Protein concentration was determined by the BCA assay. Equal amounts of protein (20–30 µg) were mixed with 4× Laemmli buffer (with 5% β-mercaptoethanol) and denatured at 95 °C for 5 min. Proteins were separated by SDS–PAGE and transferred to PVDF membranes (0.45 µm; 100 V, 60–90 min). Membranes were blocked at room temperature for 1 h in 5% nonfat milk or 5% BSA in TBS-T, incubated with primary antibodies (USP5, PTRF, ZDHHC3, β-Actin, etc.) overnight at 4 °C, washed, and then incubated with HRP-conjugated secondary antibodies for 1 h at room temperature. Signals were detected by ECL and imaged; band intensities were quantified in ImageJ and normalized to β-Actin. All experiments were performed in at least three independent replicates.

Primary antibodies: USP5 (Proteintech, 10473-1-AP), PTRF (Proteintech, 18892-1-AP), ZDHHC3 (Santa Cruz, sc-377378), β-Actin (Proteintech, 66009-1-Ig), Flag (#14793, Cell Signaling Technology), Myc (#2276, CST), HA (#3724, CST), GST (#2624, CST). All antibodies were used at manufacturer-recommended dilutions.

### Co-immunoprecipitation (Co-IP)

Cells were collected 36–48 h after transfection or treatment. For ubiquitination-related Co-IP, 10 µM MG132 was added 4–6 h before harvest to inhibit proteasomal degradation.

Native Co-IP: After two PBS washes, cells were lysed in IP buffer (with N-ethylmaleimide, NEM). Cleared lysates were precleared with Protein A/G magnetic beads for 30 min, then incubated with specific antibodies or isotype IgG at 4 °C overnight with rotation, followed by 1–2 h incubation with Protein A/G beads the next day. Beads were washed 3–5 times with buffer containing or lacking high salt (300 mM NaCl) to reduce nonspecific binding, and bound proteins were eluted by boiling for Western blotting.

Denaturing Co-IP: Cells were lysed in 1% SDS lysis buffer and heated at 95 °C for 5 min; SDS concentration was then diluted to ≤0.1%, and subsequent steps were as per native Co-IP. Each experiment included input and IgG controls, and reciprocal Co-IP was performed when feasible.

### Protein stability assay (CHX chase)

To assess PTRF stability under different conditions, USP5 was knocked down with specific shRNAs in A375 and SK-MEL-28 cells (non-targeting shRNA as control); in HEK-293T cells, groups included Mock, USP5-WT (overexpression), and USP5-C335A (catalytically inactive). Cycloheximide (CHX) at 50 µg/mL—validated to effectively block translation without acute cytotoxicity—was added to inhibit de novo protein synthesis. Cells were harvested at 0, 3, 6, and 9 h for Western blot analysis of PTRF, with β-Actin as loading control. Normalized band intensities were used to fit half-life curves and compare PTRF degradation rates across conditions.

### Click chemistry and acyl-PEG exchange (APE)

Palmitoylation detection: S-palmitoylation of USP5 was examined using metabolic labeling with 17-ODYA (17-octadecynoic acid, Sigma) combined with click chemistry. Cells were incubated in medium containing 50 µM 17-ODYA for 16 h to incorporate alkyne-palmitate into newly synthesized proteins. After lysis (50 mM Tris-HCl pH 7.5, 150 mM NaCl, 0.5% NP-40, protease inhibitors) and clarification, click reactions (Click-iT reagents: CuSO₄, TCEP, TBTA, and biotin-azide) were performed at 37 °C for 1 h to conjugate alkyne groups to azide-biotin. Biotinylated proteins were enriched with streptavidin magnetic beads and analyzed by Western blot. Hydroxylamine (HAM) pretreatment served as a negative control to verify modification specificity.

APE assay: Endogenous USP5 palmitoylation was validated by acyl-PEG exchange. After lysis, samples were treated with hydroxylamine (0.7 M, pH 7.4) to cleave thioester bonds and expose thiols, followed by labeling with mPEG-maleimide (5 kDa, Sigma). USP5 band upshift indicated the number of palmitoylation sites. Input and hydroxylamine-minus controls were processed in parallel to ensure specificity.

### Subcutaneous xenograft model

Male BALB/c nude mice (4–6 weeks old, ~18–22 g) were obtained from an accredited animal facility and housed under SPF conditions. Control and experimental stable cell lines (2 × 10⁶ cells in 100 µL PBS:Matrigel = 1:1) were injected subcutaneously into the right axilla or dorsal flank. Tumor length (L) and width (W) were measured with calipers every 3–4 days, and volume was calculated as *V* = 0.5 × *L* × *W*². Mice were euthanized at 4 weeks or upon reaching ethical endpoints; tumors were excised, weighed, and processed for histology. All procedures complied with national guidelines for animal care and use. Excised tumor tissues were divided for parallel analyses. For biochemical measurements, a portion of each tumor was weighed and homogenized in ice-cold lysis buffer; supernatants were collected after centrifugation and normalized by total protein concentration. 4-HNE and MDA were quantified using commercial kits according to the manufacturers’ instructions, and ROS levels were measured using a fluorescence-based assay as indicated.

### Assessment of ferroptosis

Cells were treated with mechanistically distinct ferroptosis inducers, including erastin (system xc− inhibitor) and RSL3 (GPX4 inhibitor), at the indicated concentrations and durations for each experiment. Where indicated, ferrostatin-1 (Fer-1) was added as a ferroptosis inhibitor rescue control. Cell viability was determined by CCK-8 assays following drug treatment. Intracellular ROS was measured by flow cytometry using a ROS detection kit (Njjcbio, Nanjing, China), based on DCFH-DA fluorescence, according to the manufacturer’s instructions. GSH levels were measured using a GSH assay kit (Beyotime, China) with absorbance read at 412 nm. Lipid peroxidation was assessed by quantifying 4-HNE and MDA using commercial kits as specified by the manufacturers; signals were normalized to total protein concentration when applicable. For ultrastructural assessment, cells were fixed immediately after treatment in 2.5% glutaraldehyde/0.1 M buffer (pH 7.4) at 4 °C overnight, post-fixed in 1% osmium tetroxide for 1 h, dehydrated through graded ethanol, embedded in epoxy resin, sectioned (~70 nm), stained with uranyl acetate and lead citrate, and imaged at 80 kV by TEM. Mitochondrial features consistent with ferroptosis (reduced volume, diminished/absent cristae, and membrane densification) were evaluated using predefined criteria.

### Statistical analysis

Data are presented as mean ± standard deviation (SD), with all experiments performed in at least three independent replicates. Two-group comparisons used two-tailed Student’s *t* tests; for three or more groups, one-way ANOVA followed by Tukey’s post-hoc test was applied. When normality or homoscedasticity assumptions were not met, non-parametric tests were used (Mann–Whitney U or Kruskal–Wallis with Dunn’s post-hoc test). Statistical significance was set at *P* < 0.05. Data analysis and visualization were performed primarily in GraphPad Prism 9 and R (v4.2.0). For quantitative assays, individual biological replicates are displayed as dots whenever possible; “*n*” denotes independent biological replicates (cell-based experiments) or individual mice (xenografts), as specified in each figure legend. Exact statistical tests and multiple-comparison procedures used for each dataset are indicated in the corresponding figure legends.

## Results

### Bioinformatic analyses indicate that USP5 is associated with melanoma cell proliferation

**Fig. 1:**
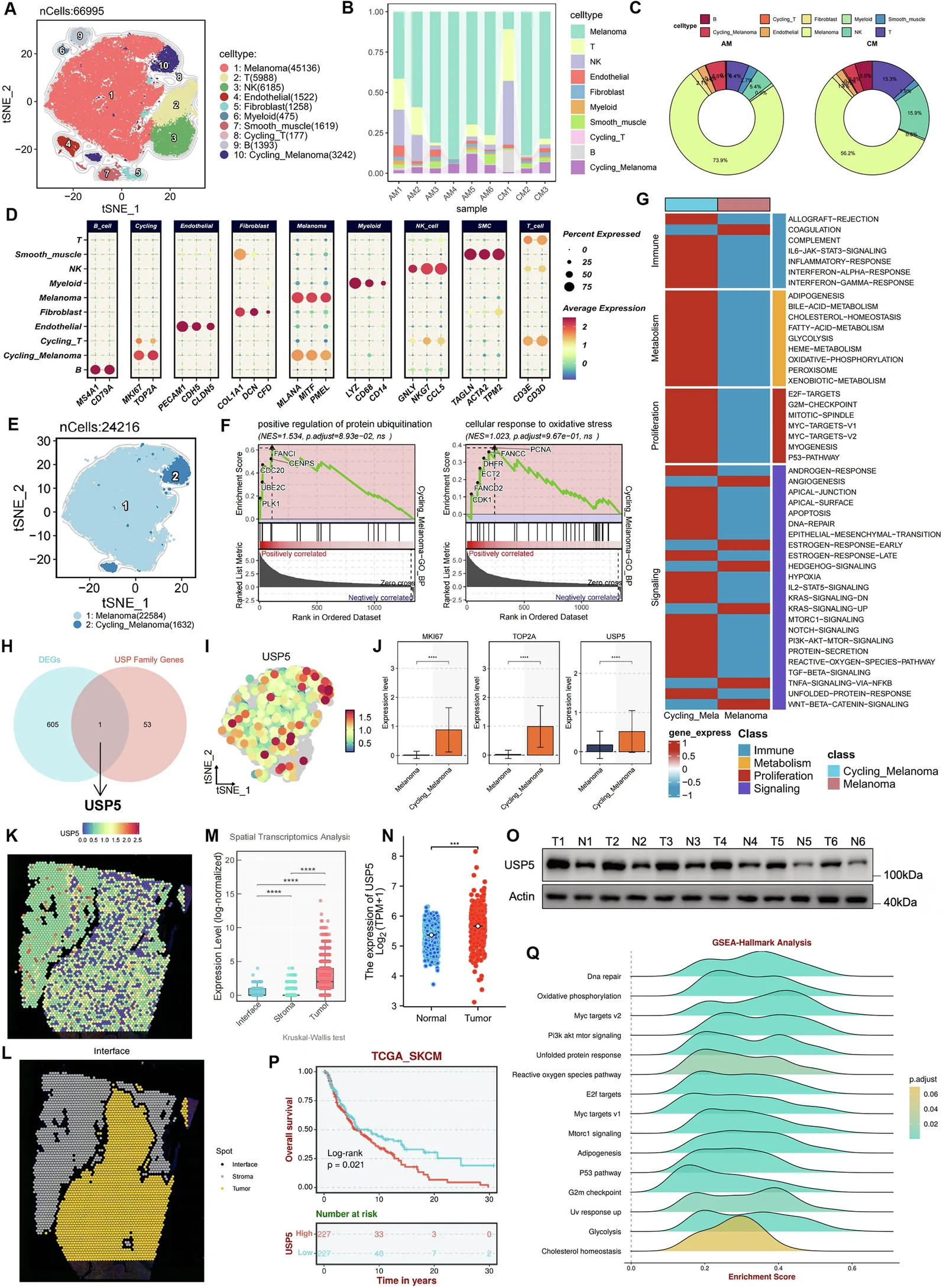
Single-cell and spatial transcriptomic analyses identify a proliferative melanoma subpopulation and implicate a potential role for USP5. **A** t-SNE visualization of integrated, quality-controlled cells from 6 acral melanoma (AM) and 3 cutaneous melanoma (CM) samples (GSE215120), annotated into 10 major cell types. **B**, **C** Relative proportions of each cell type across individual samples and between AM/CM groups (bar and donut charts), quantifying differences in tumor microenvironment composition. **D** Bubble plot of canonical marker gene expression across cell types; dot size reflects the fraction of cells, and color (Z-score) indicates relative expression. **E** Zoomed t-SNE view showing the distribution of malignant melanoma cells and proliferative melanoma cells (Cycling_Melanoma). **F** GSEA showing enrichment of ubiquitination- and oxidative stress–related signatures in Cycling_Melanoma compared with malignant melanoma cells (NES and *P* values shown). **G** GSVA pathway scores indicating positive associations between Cycling_Melanoma and pathways related to immunity, metabolism, and proliferation (heatmap of normalized pathway scores). **H** Intersection of differentially expressed genes between malignant melanoma cells and Cycling_Melanoma with 54 USP family members; USP5 is the only overlapping candidate. **I** Feature plot showing the distribution of USP5 expression in melanoma cells. (J) Violin plots comparing TOP2A, MKI67, and USP5 expression across melanoma subclusters, highlighting upregulation in Cycling_Melanoma. (K) Spatial transcriptomics section showing the spatial expression pattern of USP5 in melanoma tissue. **L**, **M** SpaCET-based cell composition inference and regional segmentation (tumor, stroma, boundary) indicating that USP5 signals are enriched in the tumor core. **N** Combined TCGA-SKCM and GTEx analysis comparing USP5 expression between tumor and normal samples. **O** Western blot analysis of USP5 protein levels in paired melanoma and adjacent non-tumor tissues (*n* = 6 pairs). **P** Kaplan–Meier overall survival analysis of the TCGA-SKCM cohort stratified by USP5 expression (cutoff as indicated). (Q) GSEA of Hallmark gene sets in the TCGA cohort indicates associations of USP5 with oxidative phosphorylation, reactive oxygen species (ROS) pathways, and DNA repair.

To investigate tumor heterogeneity in melanoma, we analyzed single-cell RNA-sequencing data from GSE215120, comprising six acral melanoma (AM) and three cutaneous melanoma (CM) samples. Ten major cell types were identified across all samples (Fig. 1A), with differing proportions between AM and CM (Fig. 1B, C). Based on canonical marker gene expression, we annotated the principal populations: melanoma cells (DCT, MLANA, PMEL), endothelial cells (PECAM1, VWF), fibroblasts (DCN, COL3A1, CFD), smooth muscle cells (TAGLN, ACTA2, TPM2), B cells (MS4A1, CD79A), myeloid cells (LYZ, CD14), T cells (CD3E, CD3D), NK cells (GNLY, NKG7), and highly proliferative “Cycling” cells (TOP2A, MKI67). Cycling cells were further subdivided into proliferating T cells and proliferating melanoma cells. We visualized cell type-specific marker expression and proportions using bubble plots (Fig. 1D), and t-SNE mapping highlighted the distribution of melanoma cells versus Cycling melanoma cells (Fig. 1E). GSEA indicated that Cycling_Melanoma was enriched for protein ubiquitination and oxidative stress-related signatures, suggesting that ubiquitin pathway components and stress-response programs are transcriptionally upregulated in proliferating melanoma cells (Fig. 1F). Consistently, GSVA showed positive correlations between Cycling_Melanoma and immune-, metabolism-, and proliferation-related pathways (Fig. 1G).

Because DUB activity is strongly influenced by post-translational regulation, binding partners, and subcellular localization, these transcriptomic analyses primarily reflect expression associations rather than enzymatic activity. Genes of the USP family encode ubiquitin-specific proteases that participate in ubiquitination/deubiquitination and play essential roles in signaling, membrane trafficking, and stress responses. Our previous work demonstrated that USP5 stabilizes PD-L1 by deubiquitination, thereby promoting immune evasion in melanoma [14]. Here, differential expression analysis between melanoma cells and Cycling_Melanoma followed by intersection with the 54 USP family members identified USP5 as the sole overlapping gene (Fig. 1H). Notably, USP5 was markedly upregulated in Cycling_Melanoma (Fig. 1I, J). Spatial transcriptomics further delineated the spatial distribution of USP5 (Fig. 1K), and SpaCET inference suggested that USP5 is concentrated within tumor-core regions (Fig. 1L, M). Integrating TCGA and GTEx datasets, USP5 was broadly overexpressed in melanoma and its high expression correlated with poorer overall survival (Fig. 1N, P). Western blotting likewise confirmed higher USP5 protein levels in tumor tissues versus adjacent non-tumor controls (Fig. 1O). Finally, in Hallmark gene set GSEA of the TCGA cohort, USP5 expression was closely associated with oxidative phosphorylation, reactive oxygen species pathways, and DNA repair (Fig. 1Q).

### USP5 is highly expressed in melanoma and promotes tumor cell proliferation

**Fig. 2:**
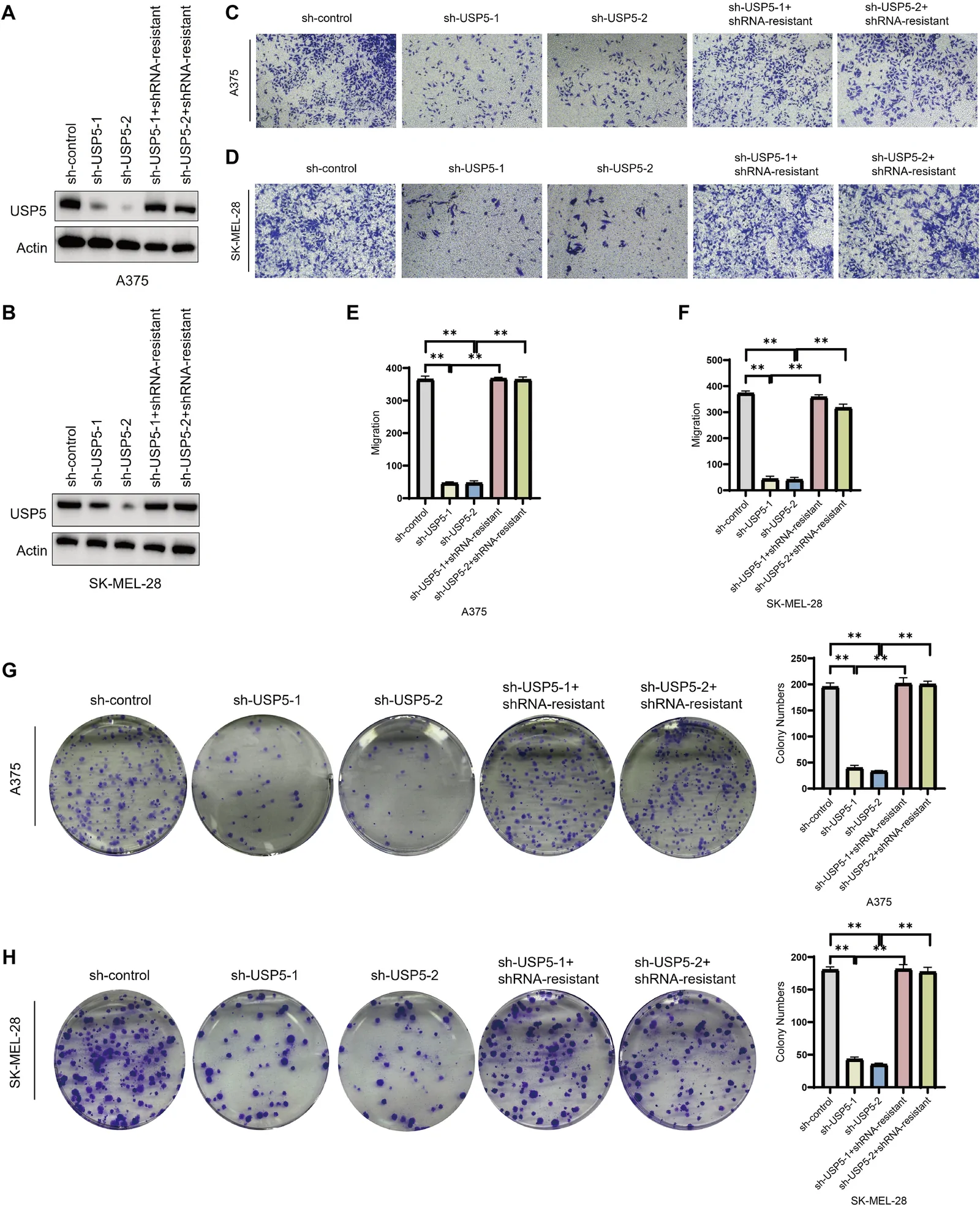
USP5 promotes melanoma cell migration and clonogenic growth, validated by genetic rescue and proliferation control. **A**, **B** Western blot verifying stable USP5 knockdown using two independent shRNAs (shUSP5-1, shUSP5-2) and restoration by shRNA-resistant USP5 re-expression in A375 and SK-MEL-28 cells; Actin serves as loading control. **C**–**F** Transwell migration assays showing that USP5 knockdown decreases migration and shRNA-resistant USP5 re-expression rescues migratory capacity in A375 (**C**, **D**) and SK-MEL-28 (**E**, **F**) cells. Cells were pretreated with mitomycin C to inhibit proliferation prior to migration assays (dose/time as indicated). Representative images and quantification (cells/field) are shown. Colony formation assays showing reduced clonogenicity upon USP5 knockdown and rescue by shRNA-resistant USP5 in A375 (**G**) and SK-MEL-28 (**H**) cells; representative wells and quantification are shown. Data are shown as mean ± SD from independent biological replicates (*n* = 3). Statistical tests are indicated in panels.

To define the biological function of USP5 and to exclude potential off-target effects, we established stable USP5 knockdown models in A375 and SK-MEL-28 cells using two independent lentiviral shRNAs (shUSP5-1 and shUSP5-2), and performed genetic rescue by re-expressing an shRNA-resistant USP5 construct. Western blotting confirmed that both shRNAs efficiently reduced USP5 protein levels, whereas shRNA-resistant re-expression restored USP5 abundance in the corresponding knockdown backgrounds (Fig. 2A, B).In transwell migration assays, depletion of USP5 markedly decreased the number of migrated cells in both A375 and SK-MEL-28 lines, while reintroduction of shRNA-resistant USP5 largely rescued the migration defect (Fig. 2C–F). To minimize potential confounding from proliferation, cells were pretreated with mitomycin C prior to migration assays; under these proliferation-inhibited conditions, USP5 knockdown still significantly reduced migration, and shRNA-resistant USP5 re-expression restored migratory capacity, supporting a motility-intrinsic role of USP5. Consistent with these findings, colony formation assays showed that USP5 knockdown significantly impaired clonogenic growth, and this reduction was substantially reversed upon shRNA-resistant USP5 re-expression (Fig. 2G, H). Together, the concordant phenotypes observed with two independent shRNAs and their restoration by genetic rescue support a specific, cell-intrinsic requirement of USP5 for efficient melanoma cell migration and clonogenic outgrowth in vitro.

### USP5 specifically binds its substrate PTRF

**Fig. 3:**
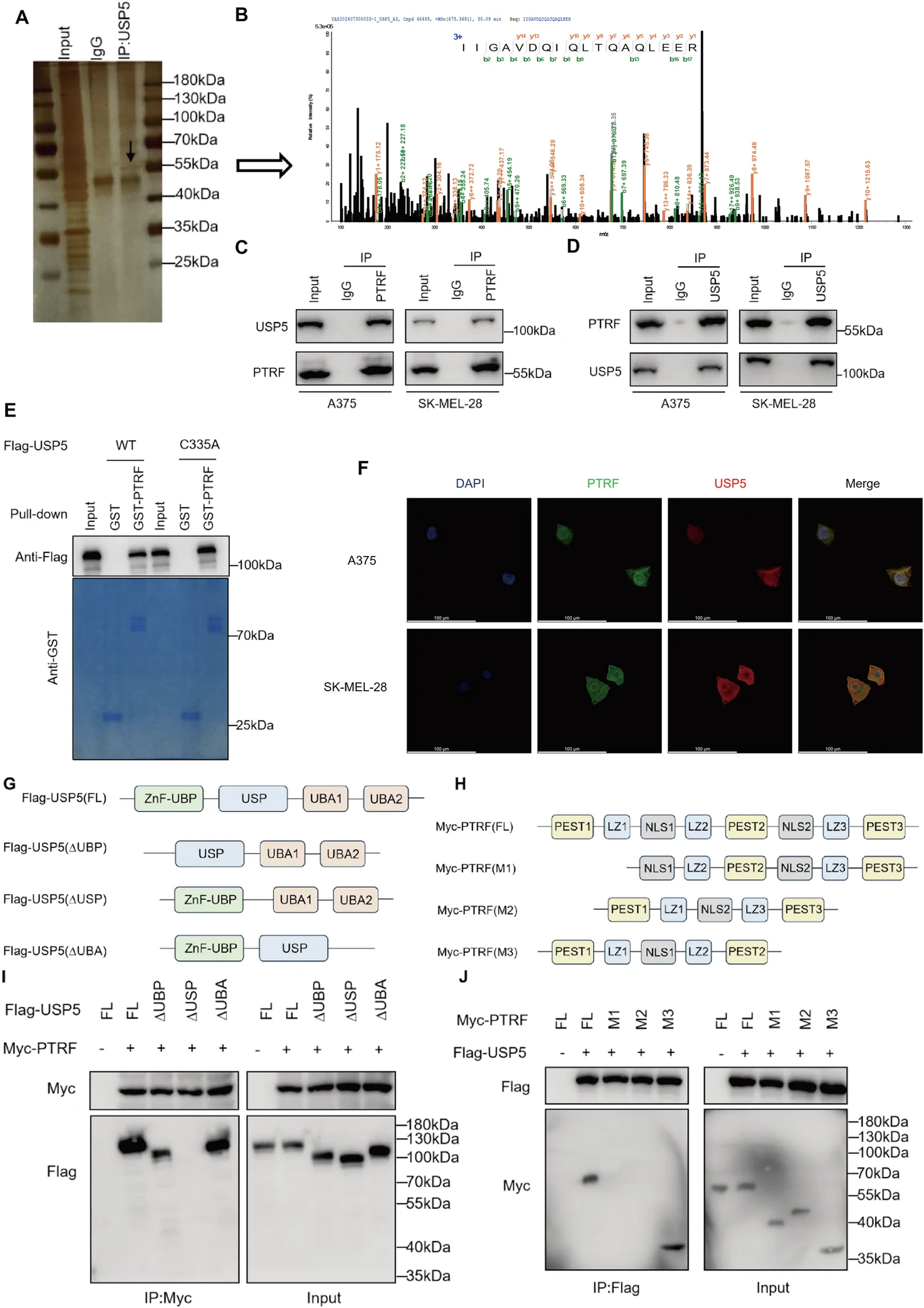
USP5 interacts with PTRF and stabilizes its function in melanoma cells. **A** Silver staining following USP5 pull-down reveals a band at the expected molecular weight of PTRF. **B** Mass spectrometry data confirm PTRF as a putative USP5 substrate. **C** Co-immunoprecipitation (co-IP) experiments in A375 and SK-MEL-28 cells confirm the interaction between endogenous USP5 and PTRF. **D** Endogenous forward and reverse co-IPs from A375 and SK-MEL-28 cells further validate the USP5–PTRF binding. **E** GST pull-down assays demonstrate that purified GST-PTRF directly pulls down Flag-tagged USP5, regardless of whether USP5 is wild-type (WT) or the catalytically inactive C335A mutant, indicating a direct interaction. **F** Immunofluorescence images showing colocalization of USP5 (red) and PTRF (green) in A375 and SK-MEL-28 cells, confirming their interaction at the subcellular level. Scale bar = 100 μm. **G** Schematic diagram of USP5 truncation constructs (FL: full-length, ΔUBP: missing UBP domain, ΔUSP: missing USP domain, ΔUBA: missing UBA domain). **H** Schematic diagram of PTRF truncation constructs (FL: full-length, M1: deletion of PEST1, M2: deletion of L1, M3: deletion of L2). **I** Co-IP results show that Myc-PTRF-FL and Myc-PTRF-M3, but not other PTRF truncation mutants, co-precipitate with Flag-USP5, indicating that the M3 region of PTRF is critical for binding. **J** Co-IP experiments with USP5 truncation mutants demonstrate that the USP domain of USP5 is essential for binding to PTRF, as the Flag-USP5-ΔUSP mutant failed to co-precipitate with PTRF.

To investigate the molecular mechanism by which USP5 enhances melanoma aggressiveness, we first identified USP5-interacting proteins using LC-MS/MS (Supplementary Table 2). Silver staining of the USP5 pull-down assay revealed a band at the expected molecular weight of PTRF (Fig. 3A). Mass spectrometry further confirmed PTRF as a potential USP5 substrate (Fig. 3B). To validate this interaction, we performed co-immunoprecipitation (co-IP) experiments in A375 and SK-MEL-28 cells, which demonstrated robust binding between endogenous USP5 and PTRF (Fig. 3C, D).

Next, we performed GST pull-down assays to confirm the direct interaction between USP5 and PTRF. The results showed that purified GST-PTRF could pull down Flag-tagged USP5, irrespective of whether USP5 was wild-type (WT) or the catalytically inactive C335A mutant, indicating a direct and stable interaction between these two proteins (Fig. 3E). To further explore their subcellular interaction, immunofluorescence experiments were conducted in A375 and SK-MEL-28 cells. Immunofluorescence showed partial spatial overlap of USP5 and PTRF signals in A375 and SK-MEL-28 cells, consistent with their interaction (Fig. 3F).

To map the interaction interfaces between USP5 and PTRF, we generated truncation mutants of both proteins. USP5 constructs included full-length USP5 (FL), as well as deletion mutants missing the UBP, USP, or UBA domains (Fig. 3G). PTRF constructs included full-length PTRF (FL), and truncated versions with deletions in the PEST, NLS, and LZ domains (M1, M2, and M3) (Fig. 3H). Co-IP assays in HEK293T cells revealed that only Myc-PTRF-FL and Myc-PTRF-M3, but not other truncation mutants, co-precipitated with Flag-USP5, indicating that the M3 region of PTRF is critical for the USP5–PTRF interaction (Fig. 3I). In contrast, the USP5-ΔUSP mutant, which lacks the USP domain, failed to bind PTRF, confirming the essential role of the USP domain in the interaction (Fig. 3J). These results establish a direct interaction between USP5 and PTRF, and they further suggest that the USP domain of USP5 and the M3 region of PTRF are necessary for their binding.

### USP5 maintains PTRF protein stability

**Fig. 4:**
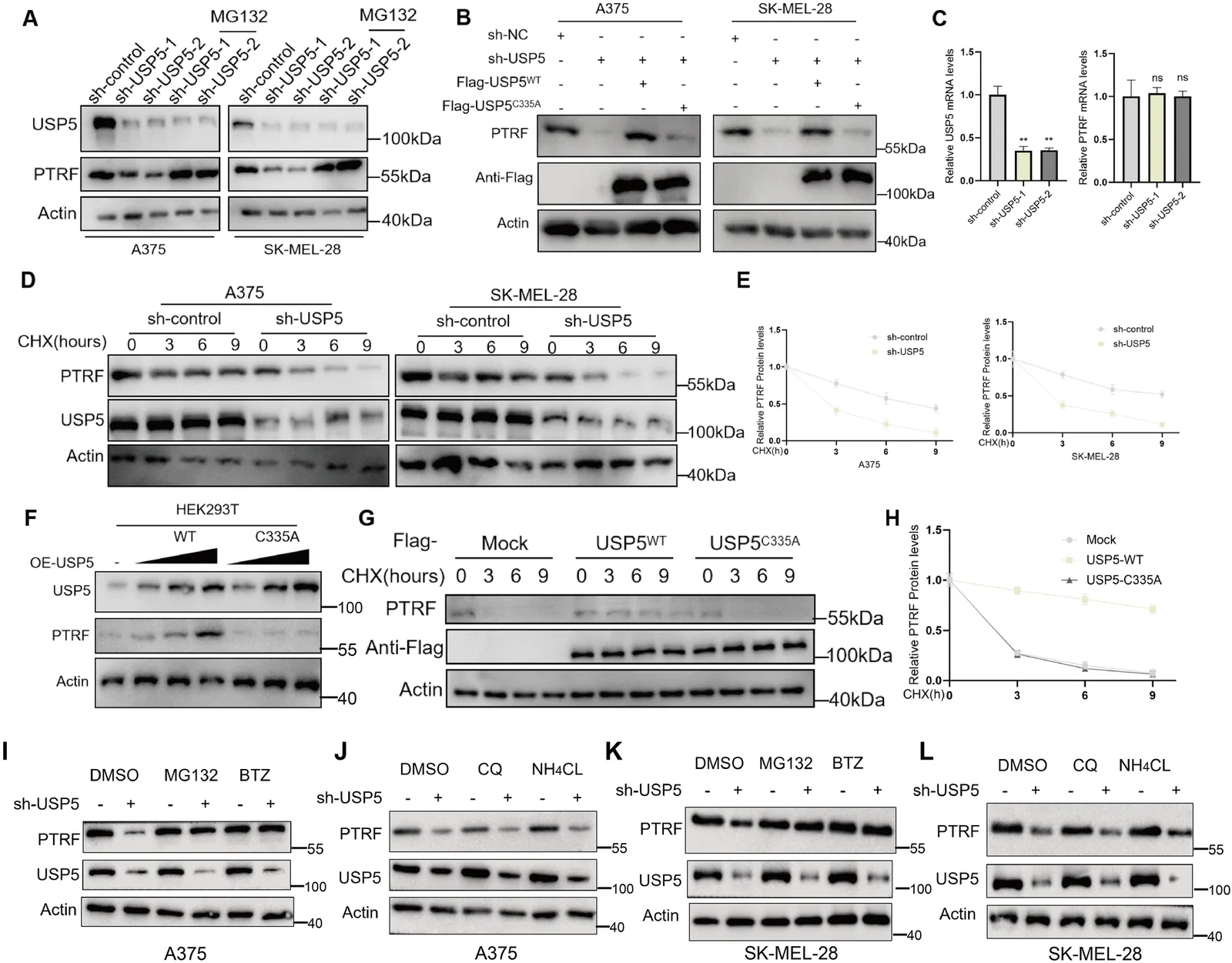
USP5 maintains PTRF protein stability via proteasome-dependent degradation control. **A** USP5 knockdown reduces PTRF protein levels in A375 and SK-MEL-28 cells, which is restored by MG132 (dose/time as indicated). **B** Re-expression of shRNA-resistant USP5-WT, but not the catalytically inactive C335A mutant, restores PTRF protein levels in USP5-depleted A375 and SK-MEL-28 cells. **C** qRT–PCR showing that USP5 knockdown does not significantly alter PTRF mRNA (normalized to ACTB; *n* = 3). (D, E) CHX chase assays showing accelerated PTRF degradation upon USP5 depletion (**D**) with quantification of PTRF half-life (**E**). **F** In HEK293T cells, USP5-WT—but not C335A—elevates PTRF protein levels in a dose-dependent manner. CHX chase assays showing that USP5-WT—but not C335A—enhances PTRF stability over time (**G**) with quantification (**H**). **I**, **J** In A375 cells, proteasome inhibitors (MG132 or BTZ) rescue PTRF reduction caused by USP5 knockdown (**I**), whereas lysosome/autophagy inhibitors (CQ or NH4Cl) do not (**J**). **K**, **L** Corresponding inhibitor experiments in SK-MEL-28 cells. Data are presented as mean ± SD from independent experiments (*n* = 3). Statistical tests are indicated in panels.

To define the functional significance of the USP5–PTRF axis, we silenced USP5 in A375 and SK-MEL-28 cells and observed a marked decrease in PTRF protein. This reduction was rescued by the proteasome inhibitor MG132 in both cell lines (Fig. 4A). Next, we performed re-expression experiments in USP5-depleted cells and found that shRNA-resistant USP5-WT, but not the catalytically inactive C335A mutant, restored PTRF protein abundance (Fig. 4B). At the transcript level, qPCR analysis showed that USP5 knockdown significantly reduced USP5 mRNA but did not change PTRF mRNA, indicating that USP5 regulates PTRF predominantly in a post-transcriptional manner (Fig. 4C). To directly assess PTRF protein stability, CHX chase assays demonstrated that USP5 depletion accelerated PTRF turnover in both A375 and SK-MEL-28 cells (Fig. 4D), which was quantified as a shortened PTRF half-life (Fig. 4E). In HEK293T cells, ectopic expression of USP5-WT increased PTRF protein in a dose-dependent manner, whereas USP5-C335A failed to do so (Fig. 4F). Consistently, CHX chase experiments further showed that USP5-WT, but not C335A, stabilized PTRF protein over time (Fig. 4G), as quantified by the corresponding decay curves (Fig. 4H). To determine the degradation pathway responsible for PTRF loss upon USP5 knockdown, treatment with the proteasome inhibitors MG132 or bortezomib (BTZ) restored PTRF levels in A375 cells (Fig. 4I), whereas lysosome/autophagy inhibition with chloroquine (CQ) or NH4Cl did not (Fig. 4J). Similar results were observed in SK-MEL-28 cells: MG132 or BTZ rescued PTRF protein reduction caused by USP5 silencing (Fig. 4K), while CQ or NH4Cl showed no appreciable restoration (Fig. 4L). Collectively, these data indicate that USP5 maintains PTRF protein stability in a deubiquitinase activity–dependent manner, thereby protecting PTRF from proteasome-mediated degradation.

### USP5 stabilizes PTRF by removing K48-linked polyubiquitin

**Fig. 5:**
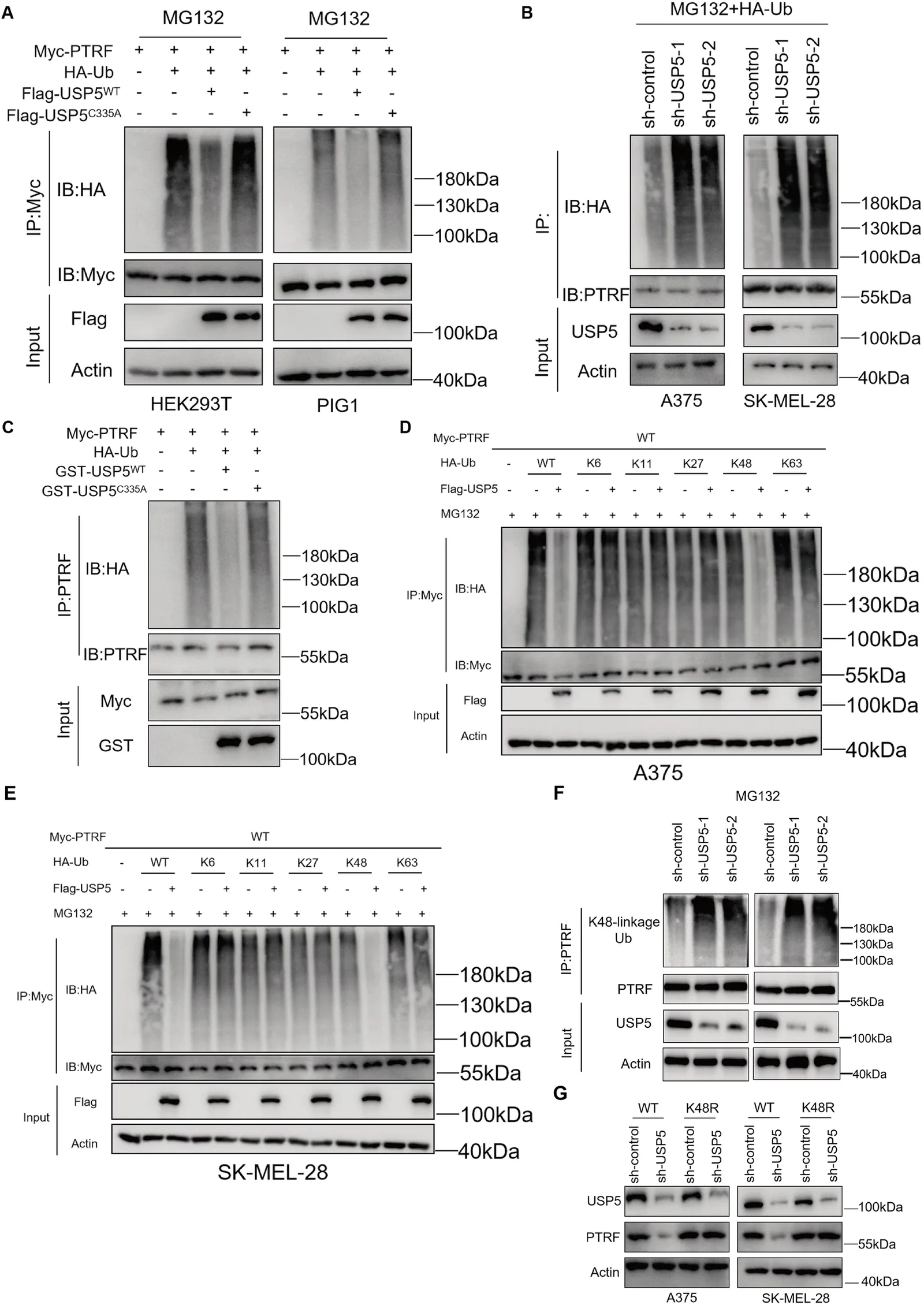
USP5 deubiquitinates PTRF and counteracts K48-linked polyubiquitin chains. **A** Ubiquitination co-IP assays in HEK293T and PIG1 cells showing that USP5-WT reduces PTRF ubiquitination, whereas USP5-C335A does not (MG132 pre-treatment as indicated). **B** USP5 knockdown increases polyubiquitination of PTRF in A375 and SK-MEL-28 cells (MG132 pre-treatment as indicated). **C** In vitro deubiquitination assay showing that purified GST–USP5-WT, but not GST–USP5–C335A, removes ubiquitin chains from ubiquitinated PTRF. Linkage-context assays in A375 (**D**) and SK-MEL-28 (**E**) cells using ubiquitin linkage mutants/restriction constructs, showing that USP5 most prominently reduces PTRF-associated ubiquitination under K48-permissive conditions. **F** Co-IP of PTRF followed by immunoblotting with a K48-linkage-specific ubiquitin antibody showing increased K48-linked ubiquitination associated with PTRF upon USP5 knockdown. **G** Functional assay using the K48R ubiquitin mutant background showing that K48R attenuates the reduction of PTRF protein caused by USP5 depletion. Representative results are from at least three independent experiments.

We next investigated whether USP5 stabilizes PTRF through deubiquitination. In ubiquitination co-immunoprecipitation assays performed in HEK293T and normal melanocyte PIG1 cells under MG132 treatment, ectopic expression of USP5-WT markedly reduced the ubiquitination signal on Myc-PTRF, whereas the catalytically inactive C335 A mutant failed to do so (Fig. 5A). Conversely, shRNA-mediated USP5 depletion in A375 and SK-MEL-28 cells increased polyubiquitination of PTRF (Fig. 5B). To determine whether USP5 can directly deubiquitinate PTRF, ubiquitinated PTRF was incubated with purified GST-USP5 proteins in a cell-free reaction; GST-USP5-WT, but not GST-USP5-C335A, efficiently removed ubiquitin chains from PTRF (Fig. 5C), supporting a direct, catalytic activity–dependent deubiquitinating effect. To explore the linkage context potentially targeted by USP5, we utilized ubiquitin linkage mutant/restriction constructs in melanoma cells and found that USP5 expression reduced PTRF-associated ubiquitination most prominently under the K48-permissive condition compared with other linkage settings (Fig. 5D, E). Consistently, co-immunoprecipitation of PTRF followed by immunoblotting with a K48-linkage–specific ubiquitin antibody showed that USP5 knockdown increased K48-linked ubiquitination associated with PTRF (Fig. 5F). Functionally, expression of the K48R ubiquitin mutant attenuated the reduction of PTRF protein caused by USP5 depletion (Fig. 5G). Collectively, these results demonstrate that USP5 can deubiquitinate PTRF and are consistent with a model in which USP5 counteracts K48-linked ubiquitination to maintain PTRF protein stability.

### S-palmitoylation of USP5 enhances its stability

**Fig. 6:**
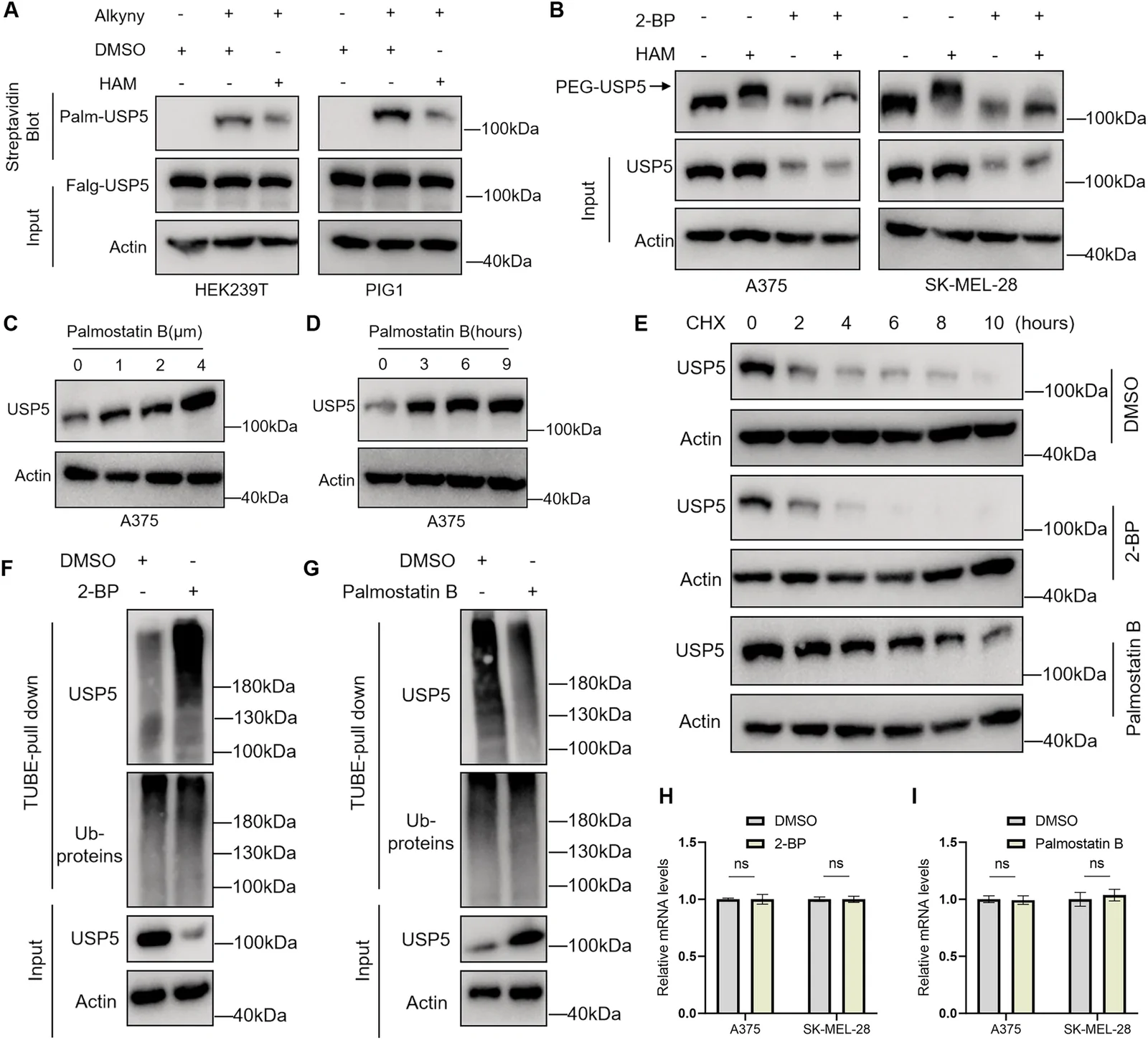
S-palmitoylation enhances USP5 stability by reducing ubiquitination. **A** Alk-C16 (17-ODYA) metabolic labeling and click chemistry detecting USP5 palmitoylation in HEK293T and PIG1 cells; hydroxylamine (HAM) abolishes the signal, confirming thioester linkage. **B** Acyl–PEG exchange (APE) assays detecting endogenous USP5 palmitoylation in A375 and SK-MEL-28 cells; untreated and HAM-treated controls are included. **C**, **D** Palmostatin B increases USP5 protein abundance in A375 cells in a dose-dependent manner (representative blots and quantification). **E** CHX chase assays showing accelerated USP5 degradation with 2-bromopalmitate (2-BP) and delayed degradation with Palmostatin B in A375 and SK-MEL-28 cells. **F**, **G** TUBE pull-down assays showing increased USP5 ubiquitination upon 2-BP treatment and decreased ubiquitination upon Palmostatin B treatment. **H**, **I** qRT–PCR showing that 2-BP and Palmostatin B do not significantly change USP5 mRNA levels. Data are presented as mean ± SD (*n* = 3). Statistical tests are indicated in panels.

We first examined whether USP5 undergoes S-palmitoylation. Using Alk-C16 metabolic labeling combined with click chemistry, we detected robust palmitoylation of USP5 in HEK293T and PIG1 cells; this signal was abolished by hydroxylamine, confirming the thioester linkage (Fig. 6A). Acyl-PEG exchange (APE) assays in A375 and SK-MEL-28 further verified endogenous USP5 palmitoylation (Fig. 6B). Functionally, the palmitoylation enhancer Palmostatin B increased USP5 protein in A375 cells in a dose-dependent manner (Fig. 6C, D). CHX chases showed that 2-bromopalmitate (2-BP) accelerated USP5 degradation, whereas Palmostatin B delayed it (Fig. 6E). Mechanistically, 2-BP treatment elevated USP5 ubiquitination, while Palmostatin B reduced ubiquitination and increased protein abundance (Fig. 6F, G). qPCR indicated that neither 2-BP nor Palmostatin B affected USP5 mRNA (Fig. 6H, I), suggesting that palmitoylation stabilizes USP5 post-translationally by suppressing ubiquitin-mediated degradation.

### ZDHHC3-associated palmitoylation reduces USP5 ubiquitination and is linked to lysosome-associated turnover

**Fig. 7:**
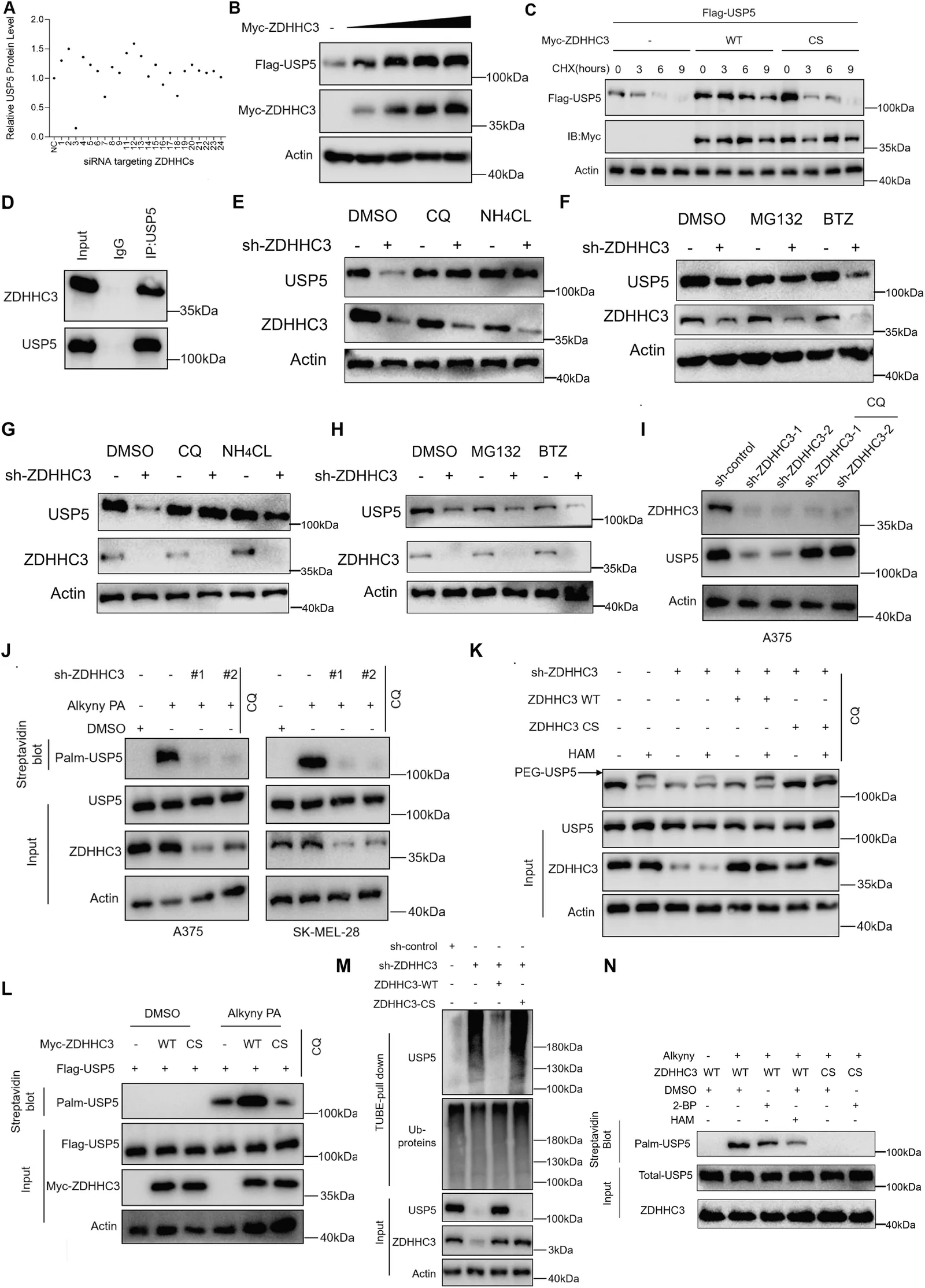
ZDHHC3 is implicated as a major regulator of USP5 palmitoylation and stability. **A** siRNA screen of ZDHHC family members identifying ZDHHC3 knockdown as markedly reducing USP5 protein abundance (quantification as shown). **B** Dose-dependent increase of USP5 protein upon ectopic Myc-ZDHHC3 expression (HEK293T; WB). **C** CHX chase showing that ZDHHC3-WT, but not the catalytically inactive ZDHHC3-CS mutant, prolongs USP5 half-life. **D** Endogenous co-IP confirming interaction between USP5 and ZDHHC3. In A375 cells, lysosome/autophagy inhibitors (CQ or NH4Cl) partially restore USP5 levels upon ZDHHC3 knockdown (**E**), whereas proteasome inhibitors (MG132 or BTZ) do not show comparable restoration under the conditions tested (**F**). **G**, **H** Corresponding inhibitor experiments in SK-MEL-28 cells. **I** Validation using two independent shRNAs against ZDHHC3 showing partial restoration of USP5 reduction by CQ in A375 cells. **J** Click-chemistry palmitoylation assay showing reduced palmitoylated USP5 signal upon ZDHHC3 silencing in A375 and SK-MEL-28 cells. **K** APE assay showing reduced palmitoylated fraction of USP5 upon ZDHHC3 knockdown and restoration by reintroduction of ZDHHC3-WT but not ZDHHC3-CS. **L** In HEK293T cells, ZDHHC3-WT promotes USP5 palmitoylation, whereas ZDHHC3-CS shows minimal effect. **M** TUBE pull-down assays showing increased ubiquitinated USP5 upon ZDHHC3 depletion and reduction by ZDHHC3-WT but not CS. **N** 2-BP reduces palmitoylated USP5 signal. Representative results are from at least three independent experiments; statistical tests are indicated where quantification is shown.

To explore the upstream regulator(s) of USP5 S-palmitoylation and stability, we performed an siRNA screen targeting the ZDHHC family and found that silencing ZDHHC3 produced one of the most pronounced reductions in USP5 protein abundance (Fig. 7A). Consistently, ectopic expression of Myc-ZDHHC3 increased USP5 protein levels in a dose-dependent manner (Fig. 7B). Cycloheximide (CHX) chase assays further showed that ZDHHC3-WT, but not the catalytically inactive CS mutant, prolonged USP5 protein half-life (Fig. 7C), suggesting that ZDHHC3 enzymatic activity is required for USP5 stabilization. Co-immunoprecipitation of endogenous proteins confirmed an interaction between ZDHHC3 and USP5 (Fig. 7D).

We next assessed the degradation route contributing to USP5 loss upon ZDHHC3 depletion. In A375 cells, ZDHHC3 knockdown decreased USP5 protein, which was partially restored by lysosomal inhibition (CQ or NH4Cl) (Fig. 7E), whereas proteasome inhibition (MG132 or BTZ) did not yield a comparable restoration under the conditions tested (Fig. 7F). Similar trends were observed in SK-MEL-28 cells (Fig. 7G, H). Moreover, using two independent shRNAs against ZDHHC3, CQ treatment partially rescued USP5 reduction in A375 cells (Fig. 7I), supporting a contribution of lysosome-associated turnover when ZDHHC3 is suppressed. Notably, these pharmacologic data indicate involvement of lysosomal degradation but do not directly demonstrate altered intracellular trafficking or lysosomal targeting of USP5.

To determine whether ZDHHC3 regulates USP5 palmitoylation in melanoma cells, we performed metabolic labeling/click-chemistry assays using alkynyl-palmitate. ZDHHC3 silencing reduced the palmitoylated USP5 signal in both A375 and SK-MEL-28 cells (Fig. 7J). An acyl-PEG exchange (APE) assay further confirmed that ZDHHC3 knockdown diminished the palmitoylated fraction of USP5, while reintroduction of ZDHHC3-WT—but not the CS mutant—restored it (Fig. 7K). In HEK293T cells, ZDHHC3-WT promoted Flag-USP5 palmitoylation in the alkynyl-palmitate labeling assay, whereas ZDHHC3-CS showed little effect (Fig. 7L). Functionally, TUBE pulldown assays indicated that ZDHHC3 depletion was associated with increased ubiquitinated USP5, which was reduced by ZDHHC3-WT but not the CS mutant (Fig. 7M). Finally, 2-bromopalmitate (2-BP) reduced the palmitoylated USP5 signal in the streptavidin-enriched palmitoylated fraction (Fig. 7N), providing supportive pharmacologic evidence, although this approach is not fully specific..

Collectively, these results suggest that ZDHHC3 is an important (and likely major) regulator associated with USP5 S-palmitoylation and protein stability in melanoma cells, and that loss of ZDHHC3 correlates with enhanced USP5 ubiquitination and increased lysosome-associated turnover, although direct evidence for USP5 trafficking or lysosomal localization remains to be established, and this aspect of the model should therefore be considered suggestive rather than definitive. Further confirmation of direct and site-specific catalysis—including USP5 palmitoylation-deficient mutant rescue and in vitro palmitoylation assays with purified proteins—will be required to establish mechanistic specificity.

### ZDHHC3 is implicated as a major regulator of USP5 palmitoylation at C471 and modulates ferroptosis-associated oxidative damage in vivo

**Fig. 8:**
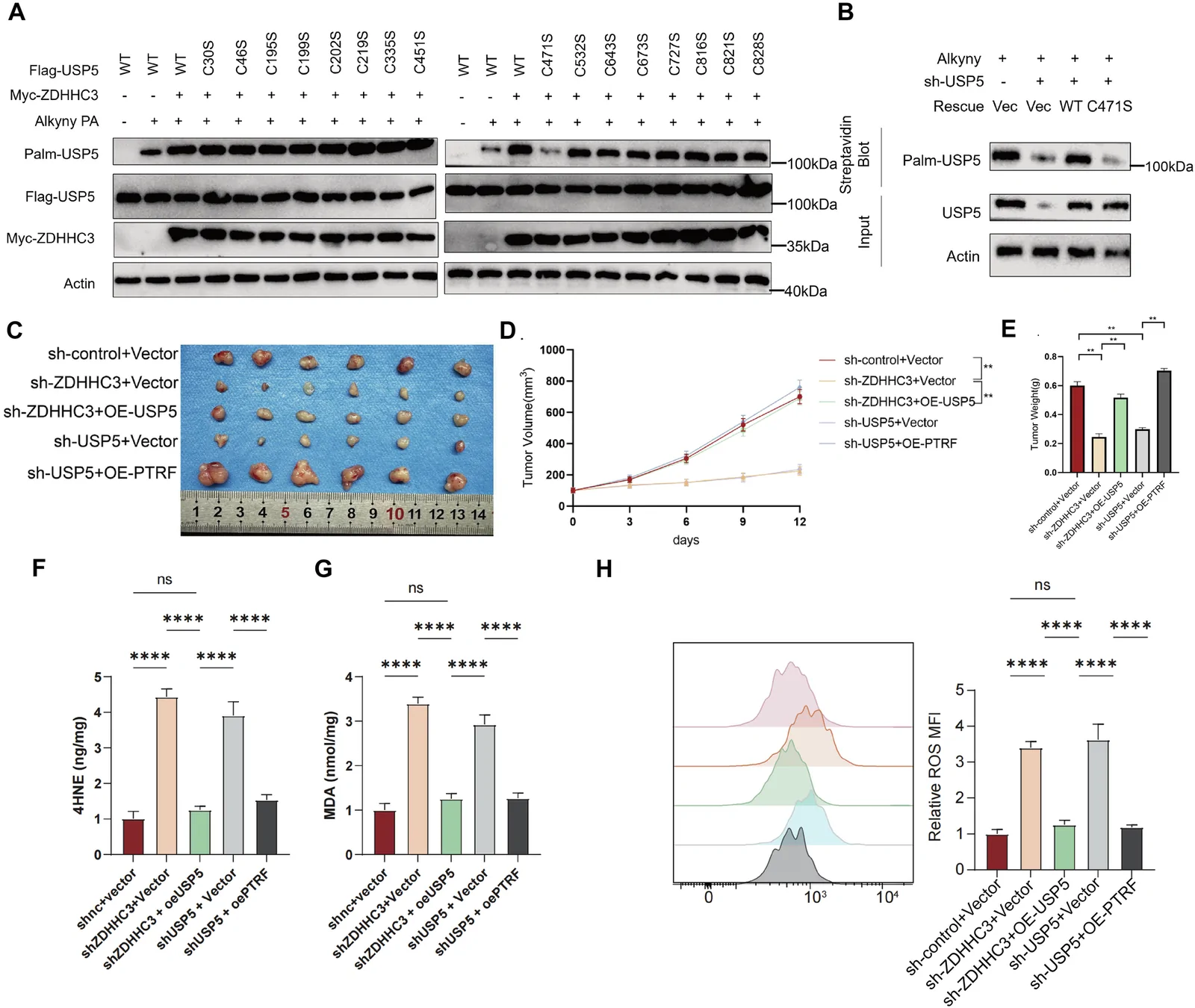
Identification of USP5 palmitoylation site C471 and palmitoylation-deficient rescue, with in vivo oxidative damage readouts. **A** Systematic cysteine-to-serine mutagenesis coupled with click-chemistry labeling identifies C471 as a major site contributing to USP5 palmitoylation (C471S markedly reduces palmitoylation signal versus WT). **B** Rescue experiment in USP5-silenced cells showing that re-expression of USP5-WT, but not the palmitoylation-deficient USP5-C471S mutant, restores USP5 palmitoylation signal. **C**–**E** Xenograft experiments showing that ZDHHC3 knockdown suppresses tumor growth and is partially reversed by USP5 overexpression; USP5 knockdown suppresses tumor growth and is partially rescued by PTRF overexpression (growth curves, representative tumors, and endpoint tumor weights as shown). Tumor tissue measurements of lipid peroxidation markers 4-HNE (**F**) and MDA (**G**) in xenografts across indicated groups. **H** Tumor tissue ROS levels across xenograft groups. Data are presented as mean ± SD (*n* = … mice/group). Statistical tests are indicated in panels.

To address whether USP5 palmitoylation is site-specific and causally linked to downstream phenotypes, we first mapped the palmitoylation site(s) on USP5. Using alkynyl-palmitate metabolic labeling with click chemistry, we performed systematic cysteine-to-serine mutagenesis of USP5 in the presence of ZDHHC3. Among the mutants tested, C471S showed a marked loss of palmitoylated USP5 signal compared with WT, indicating that C471 is a major site contributing to USP5 palmitoylation under these conditions (Fig. 8A). We then conducted rescue experiments in USP5-silenced cells: re-expression of USP5-WT, but not USP5-C471S, restored the palmitoylated USP5 signal (Fig. 8B), supporting that USP5 palmitoylation depends on C471 and providing a palmitoylation-deficient control to strengthen mechanistic specificity.

Next, we evaluated the functional relevance of the ZDHHC3–USP5–PTRF axis in vivo. In xenograft models, ZDHHC3 knockdown markedly suppressed tumor growth, which was partially reversed by USP5 overexpression; likewise, USP5 knockdown reduced tumor growth, and PTRF overexpression partially rescued this effect (Fig. 8C–E). Given the established connection between PTRF and ferroptosis-associated oxidative damage, we further measured lipid peroxidation and oxidative stress markers in tumor tissues. ZDHHC3 depletion or USP5 depletion increased 4-HNE and MDA levels, whereas USP5 or PTRF overexpression mitigated these elevations (Fig. 8F, G). Consistently, ROS levels were increased by ZDHHC3 or USP5 knockdown and were reduced upon USP5 or PTRF overexpression (Fig. 8H). Collectively, these data are consistent with a model in which ZDHHC3-dependent palmitoylation of USP5 promotes the pro-tumorigenic output of the USP5–PTRF pathway and is associated with reduced lipid peroxidation and oxidative stress markers in vivo, consistent with a potential modulation of ferroptosis-related processes rather than direct evidence of ferroptosis regulation..

### The ZDHHC3–USP5–PTRF axis modulates ferroptosis-associated phenotypes in vitro

**Fig. 9:**
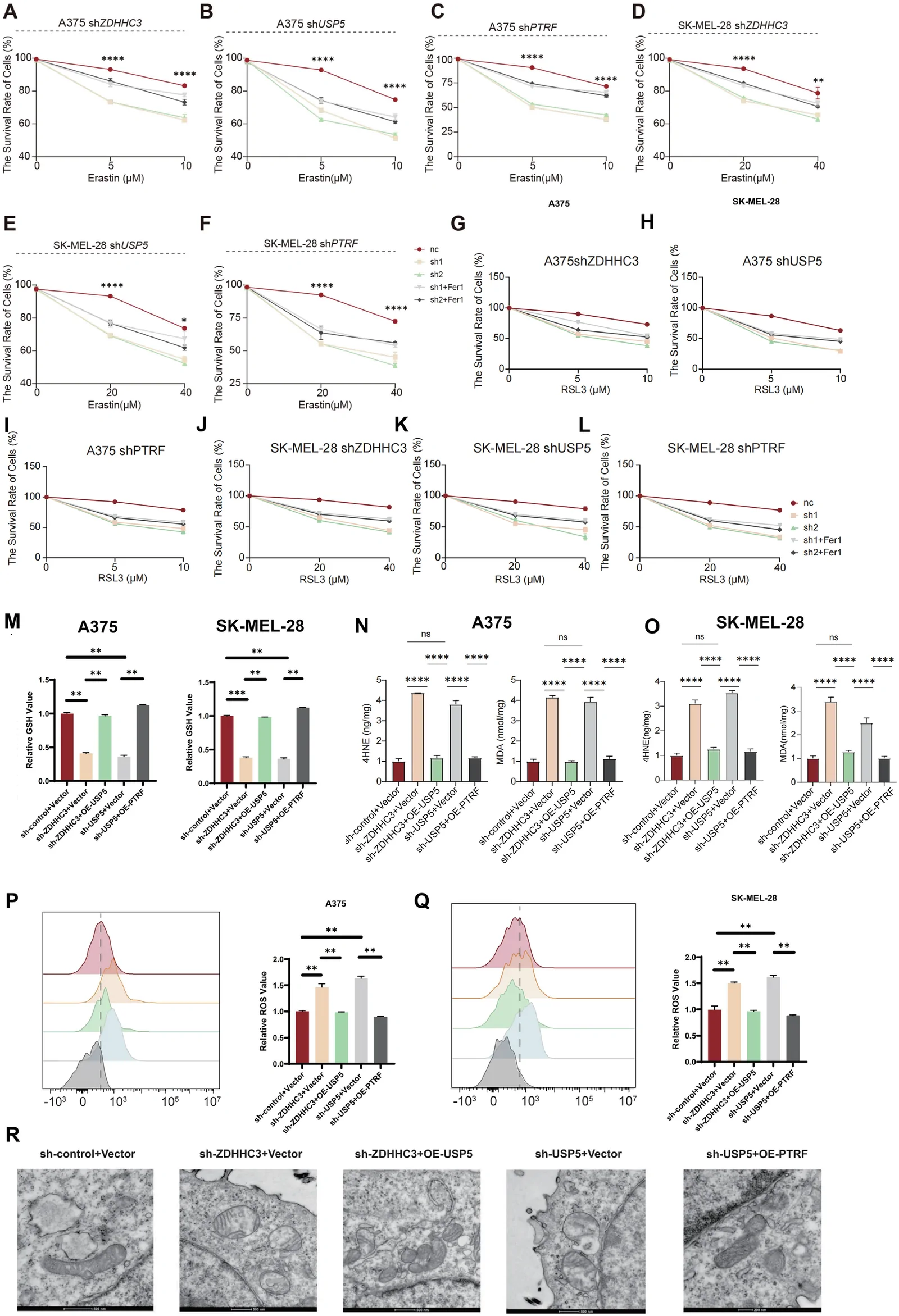
The ZDHHC3–USP5–PTRF axis modulates ferroptosis-associated phenotypes in vitro. CCK-8 dose–response assays in A375 cells treated with erastin showing increased sensitivity upon ZDHHC3 (**A**), USP5 (**B**), or PTRF (**C**) knockdown. **D**–**F** Corresponding erastin dose–response assays in SK-MEL-28 cells; ferrostatin-1 (Fer-1) rescue is shown where indicated. CCK-8 dose–response assays in A375 cells treated with RSL3 showing increased sensitivity upon ZDHHC3 (**G**), USP5 (**H**), or PTRF (**I**) knockdown. **J**–**L** Corresponding RSL3 dose–response assays in SK-MEL-28 cells; Fer-1 rescue is shown where indicated. **M** Intracellular GSH levels reduced by ZDHHC3 or USP5 knockdown and partially restored by USP5 overexpression (in ZDHHC3-silenced background) or PTRF overexpression (in USP5-silenced background), in both cell lines as indicated. **N**, **O** Lipid peroxidation markers (4-HNE and MDA) increased upon ZDHHC3 or USP5 knockdown and mitigated by USP5 or PTRF overexpression, respectively (cell lines as indicated). **P**, **Q** Flow cytometry analysis of intracellular ROS levels showing increased ROS upon ZDHHC3 or USP5 knockdown and partial reversal by USP5 or PTRF overexpression. **R** TEM images showing mitochondrial ultrastructural features consistent with ferroptosis in ZDHHC3- or USP5-depleted cells and attenuation by USP5 or PTRF overexpression (scale bar as indicated). Data are presented as mean ± SD from independent biological replicates (*n* = 3). Statistical tests are indicated in panels.

To strengthen the evidence that the observed cell death phenotype is ferroptosis-specific, we evaluated ferroptosis sensitivity using mechanistically distinct inducers and performed pharmacologic rescue. In A375 cells, silencing ZDHHC3, USP5, or PTRF increased sensitivity to the system Xc^-^ inhibitor erastin, as indicated by reduced cell survival across the tested dose range (Fig. 9A–C). Similar results were observed in SK-MEL-28 cells upon ZDHHC3, USP5, or PTRF depletion (Fig. 9D–F). To further exclude erastin-specific or non-ferroptotic effects, we repeated the assays using the GPX4 inhibitor RSL3. Knockdown of ZDHHC3, USP5, or PTRF similarly enhanced RSL3-induced loss of viability in A375 cells (Fig. 9G–I) and in SK-MEL-28 cells (Fig. 9J–L). Importantly, co-treatment with the ferroptosis inhibitor ferrostatin-1 partially restored cell viability in both erastin- and RSL3-challenged conditions, supporting that a substantial component of the observed cytotoxicity is ferroptosis-associated.

**Fig. 10:**
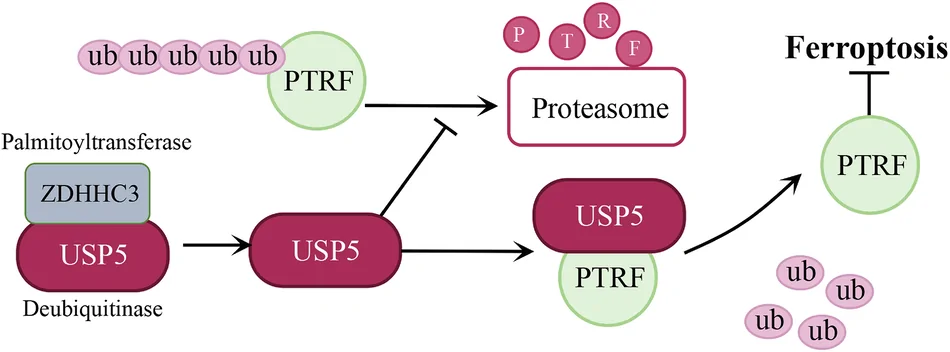
Proposed model of the ZDHHC3–USP5–PTRF axis in melanoma. Schematic summarizing the proposed mechanism: ZDHHC3 promotes USP5 S-palmitoylation (centered on C471), which is associated with increased USP5 stability. Stabilized USP5 interacts with PTRF and counteracts K48-linked ubiquitination, thereby preventing proteasome-dependent PTRF degradation and maintaining PTRF protein abundance. Elevated PTRF is associated with reduced lipid peroxidation/oxidative stress and decreased sensitivity to ferroptosis inducers, whereas loss of ZDHHC3/USP5 favors USP5 destabilization and PTRF degradation, accompanied by enhanced ferroptosis-associated phenotypes.

We next assessed canonical ferroptosis-related biochemical readouts. Consistent with increased ferroptotic vulnerability, depletion of ZDHHC3 or USP5 decreased intracellular GSH, whereas overexpression of USP5 (in the ZDHHC3-silenced background) or PTRF (in the USP5-silenced background) partially restored GSH levels in both A375 and SK-MEL-28 cells (Fig. 9M). In parallel, measurements of lipid peroxidation products showed that ZDHHC3 or USP5 knockdown increased 4-HNE and MDA, and these elevations were mitigated by USP5 or PTRF overexpression, respectively (Fig. 9N, O). Flow cytometry further demonstrated increased ROS accumulation upon ZDHHC3 or USP5 depletion, which was partially reversed by USP5 or PTRF overexpression in A375 and SK-MEL-28 cells (Fig. 9P, Q). Finally, transmission electron microscopy revealed mitochondrial ultrastructural features consistent with ferroptosis—condensed mitochondria and disrupted cristae—in ZDHHC3- or USP5-depleted cells, and these abnormalities were attenuated by USP5 or PTRF overexpression (Fig. 9R). Collectively, these data indicate that the ZDHHC3–USP5–PTRF axis regulates ferroptosis-associated cellular phenotypes in vitro, including sensitivity to ferroptosis inducers, antioxidant capacity, lipid peroxidation, and oxidative stress. Together, these findings suggest that the ZDHHC3–USP5–PTRF axis modulates ferroptosis-associated cellular phenotypes in vitro, rather than establishing a definitive mechanistic pathway(Fig. 10).

## Discussion

This study provides an in-depth investigation of a ZDHHC3–USP5–PTRF regulatory cascade in melanoma. Through a combination of molecular, biochemical, and functional approaches, we provide evidence that USP5 can contribute to PTRF protein stabilization via a deubiquitinating mechanism, and further show that USP5 itself is regulated at the post-translational level by ZDHHC3-associated S-palmitoylation. Together, these findings suggest a potential functional coupling between lipid modification and deubiquitination that may influence melanoma cell behavior under stress conditions.

Mechanistically, our data support that USP5 contributes to maintaining PTRF protein stability in a manner dependent on USP5 catalytic activity, as suggested by the loss of function observed with the catalytically inactive mutant (C335A). PTRF was initially characterized as a transcription termination factor [24], and has since been recognized as an essential structural component of caveolae [25]. Caveolae assembly requires the cavin protein family (cavin-1 to cavin-4), with cavin-1/PTRF serving as a central factor maintaining caveolar integrity and function. In breast cancer, PTRF downregulation—driven by promoter methylation—affects drug tolerance, and its loss enhances MCF7 cell sensitivity to doxorubicin [26]. In lung cancer, PTRF interacts with EGFR, suggesting a role in receptor tyrosine kinase signaling [27]. Collectively, these findings indicate that PTRF can influence caveola-dependent signaling as well as non-caveolar pathways implicated in cell migration, drug response, and tumor progression. Given the established role of EGFR in acquired drug resistance in melanoma [28], PTRF, as a key caveolar component, may contribute to melanoma aggressiveness by modulating EGFR-associated membrane signaling platforms [29].

Notably, while our experiments support a USP5–PTRF functional connection, we acknowledge that ubiquitin chain architectures on endogenous PTRF may be heterogeneous and that additional linkage-resolved approaches would further refine the mechanistic details. Importantly, given the broad substrate spectrum of USP family deubiquitinases, it is likely that USP5 regulates multiple downstream targets. While our data support PTRF as a functionally relevant substrate, it should not be interpreted as the sole or principal effector of USP5 in melanoma, and additional substrates may contribute to the observed phenotypes.

Building on this foundation, we examined the relationship between this axis and ferroptosis-associated readouts. Ferroptosis is an iron-dependent form of regulated cell death driven by lipid peroxidation and has been implicated in resistance to MAPK inhibitors and immunotherapy in melanoma. Prior studies indicate that PTRF loss disrupts membrane lipid organization, exacerbates oxidative stress, and depletes glutathione (GSH), thereby sensitizing cells to ferroptosis inducers such as Erastin [25, 30]. In our study, genetic perturbation of ZDHHC3/USP5/PTRF altered ferroptosis-associated cellular phenotypes in vitro, including sensitivity to mechanistically distinct inducers, antioxidant capacity, lipid peroxidation markers, ROS accumulation, and mitochondrial ultrastructural features.

In xenografts, manipulation of this axis affected tumor growth and was accompanied by changes in oxidative damage markers, which are consistent with altered redox and lipid peroxidation status. However, as these readouts are not entirely specific to ferroptosis, and given the complexity of redox regulation, these findings should be interpreted as supporting modulation of ferroptosis-associated phenotypes rather than establishing a definitive ferroptotic mechanism in vivo. Furthermore, these data do not directly demonstrate that tumor growth effects are ferroptosis-dependent, and additional in vivo ferroptosis-specific modulation or rescue experiments will be required to establish causality.

ZDHHC3, a member of the DHHC-CRD palmitoyltransferase family, exhibits context-dependent roles in disease. Previous reports show that ZDHHC3-mediated S-palmitoylation modulates immune regulation, metabolic homeostasis, and tumor progression [31, 32]. In various cancers, ZDHHC3 loss enhances oxidative stress and suppresses tumor viability [33], while its hyperactivation can promote immune evasion—for instance, by stabilizing PD-L1 through palmitoylation [34].

Our findings implicate ZDHHC3 as an important (and likely major) regulator associated with USP5 palmitoylation and protein stability in melanoma cells, supported by genetic perturbation, catalytic-mutant controls, and palmitoylation assays. Importantly, while inhibitor-based experiments support an involvement of lysosome-associated turnover when ZDHHC3 is suppressed, this conclusion is based on indirect pharmacological evidence, and we did not directly demonstrate altered intracellular trafficking or lysosomal targeting of USP5. Therefore, this aspect of the model should be considered suggestive rather than definitive, and additional localization-based approaches would further strengthen this part of the mechanism.

Moreover, although we mapped a major palmitoylation site and used a palmitoylation-deficient mutant to strengthen causality, in vitro palmitoylation assays with purified proteins and assessments of potential DHHC redundancy would further establish direct and site-specific catalysis.

From a therapeutic perspective, the identification of the USP5–ZDHHC3–PTRF axis provides potential intervention points in melanoma. Developing selective modulators of USP5 deubiquitinase activity or targeting palmitoylation pathways may influence PTRF stability and ferroptosis-associated phenotypes, and could potentially be explored in combination with ferroptosis-inducing strategies. However, therapeutic interpretation should be cautious: USP5 and ZDHHC3 participate in broader proteostasis and palmitoylation networks, raising potential on-target liabilities and the need for tumor-selective strategies. Previous studies have demonstrated the feasibility of targeting palmitoylation in melanoma and other cancers [35], supporting the rationale for further evaluation while carefully weighing specificity and toxicity considerations.

Naturally, this study has limitations. The precise molecular mechanisms by which PTRF regulates lipid metabolism, glutathione homeostasis, and ferroptosis-associated outcomes remain to be fully elucidated. It is also possible that USP5 stabilizes additional substrates relevant to redox regulation and stress resistance, given the broad substrate spectrum of USP family members. Furthermore, while transcriptomic analyses support clinical associations, enzymatic activity and post-translational state cannot be inferred from RNA expression alone and warrant direct measurement in patient-derived samples where feasible. Future studies integrating proteomics/ubiquitinomics and lipidomics will be essential to delineate how this pathway intersects with antioxidant defense, membrane remodeling, and therapeutic stress responses.

In summary, this study links ZDHHC3-associated S-palmitoylation of USP5 to PTRF stabilization and ferroptosis-associated cellular phenotypes, and supports a model in which this post-translational regulatory cascade may contribute to malignant features of melanoma. These findings broaden the understanding of PTM networks in melanoma and provide a framework for further mechanistic and translational validation.

## Supplementary information

Original Western blots

Primer Information

Results of Protein Mass Spectrometry

## Data Availability

Publicly available datasets were analyzed in this study. The raw data for this study were obtained from TCGA (https://xenabrowser.net/), GEO (http://www.ncbi.nlm.nih.gov/geo/) and MSigDB (https://www.gsea-msigdb.org/) databases. The MS data can be accessed in Jianguoyun Database(data link: https://www.jianguoyun.com/p/De_s42wQyvKGDhjOyaQGIAA).
