# Supplementary material for: A ZDHHC3–USP5–PTRF axis links palmitoylation to ferroptosis-associated phenotypes in melanoma
Source: Cell Death Dis. 2026 Jul 2;17(1):822. doi: 10.1038/s41419-026-09053-w (PMC13597508; doi:10.1038/s41419-026-09053-w)

Figure 1O

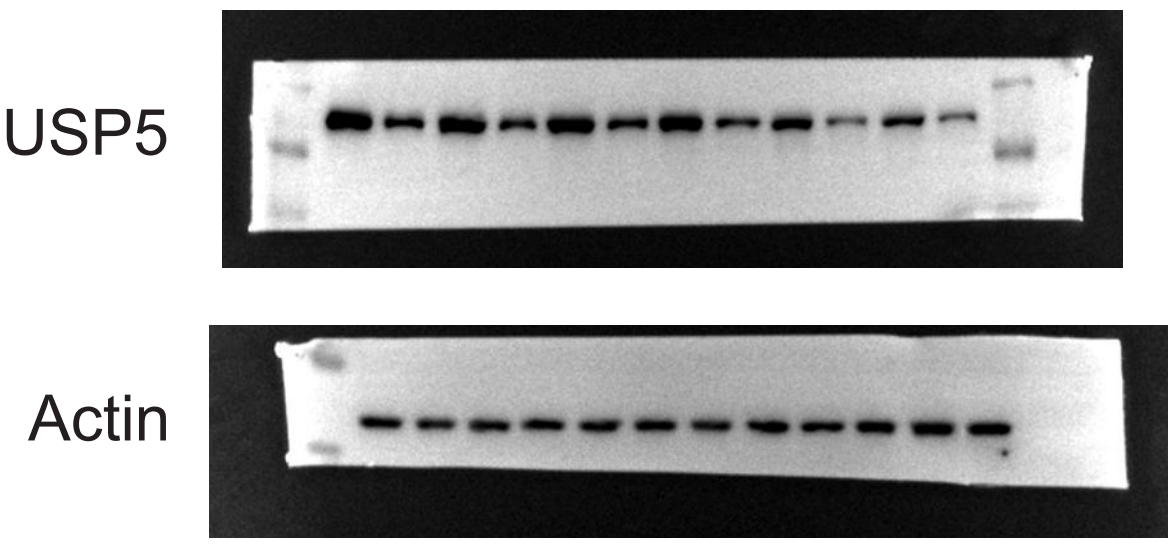

Figure 2A

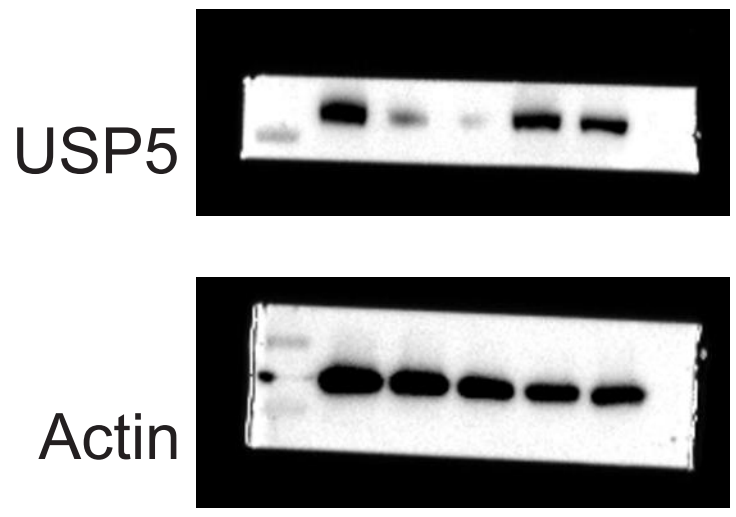

Figure 2B

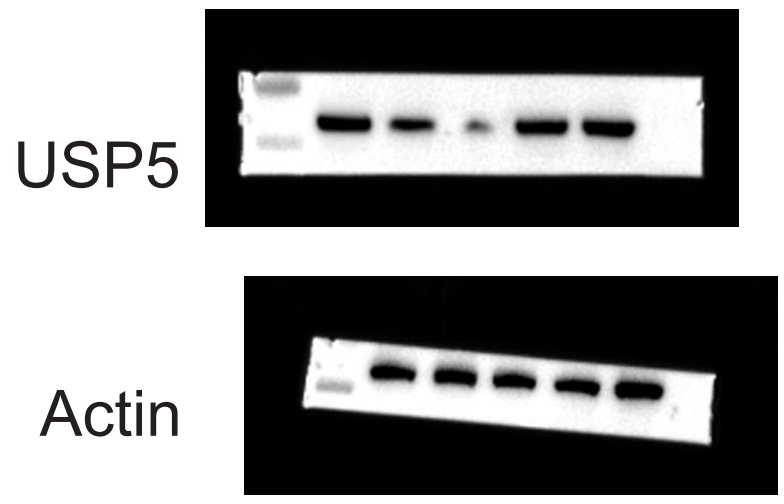

Figure 3C

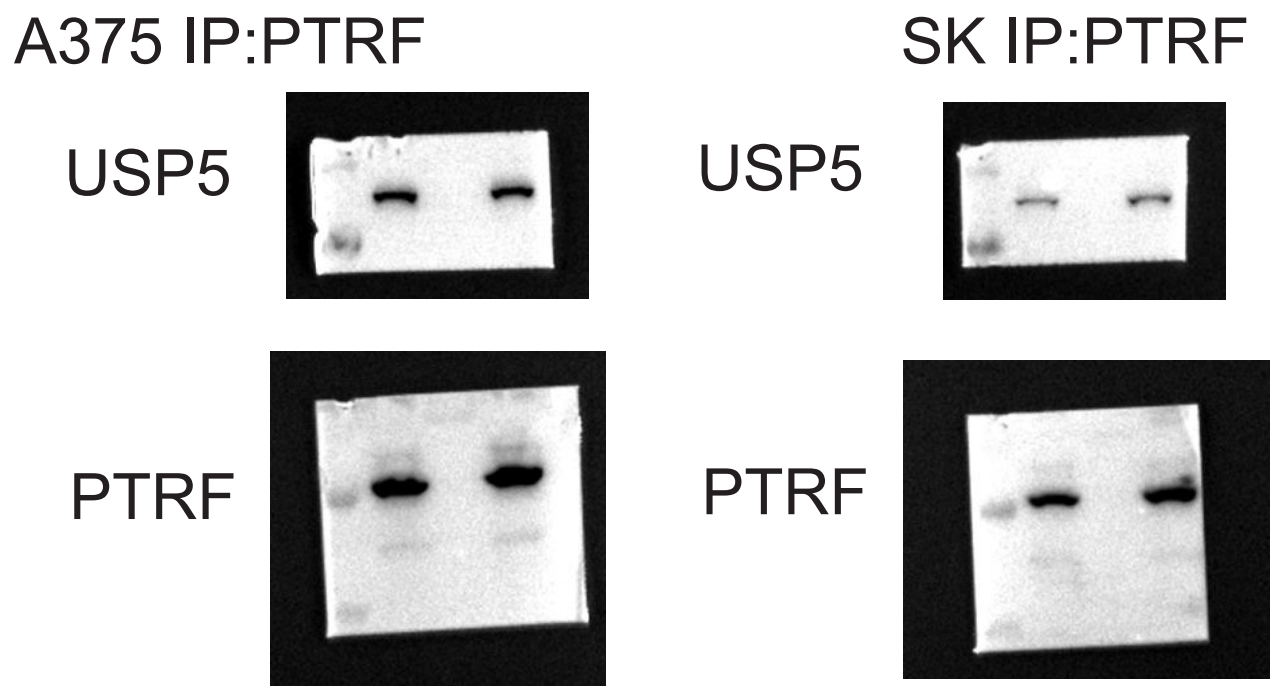

Figure 3D

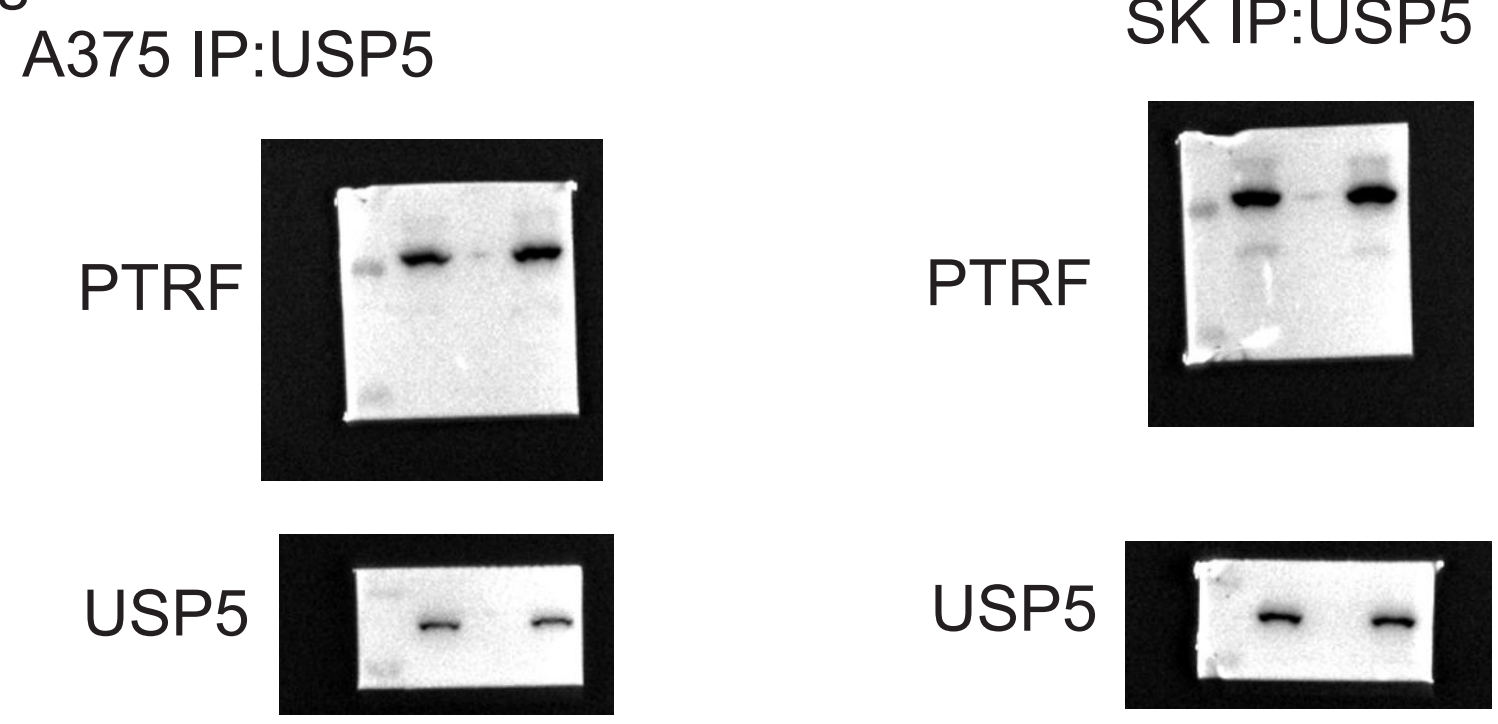

Figure 3E

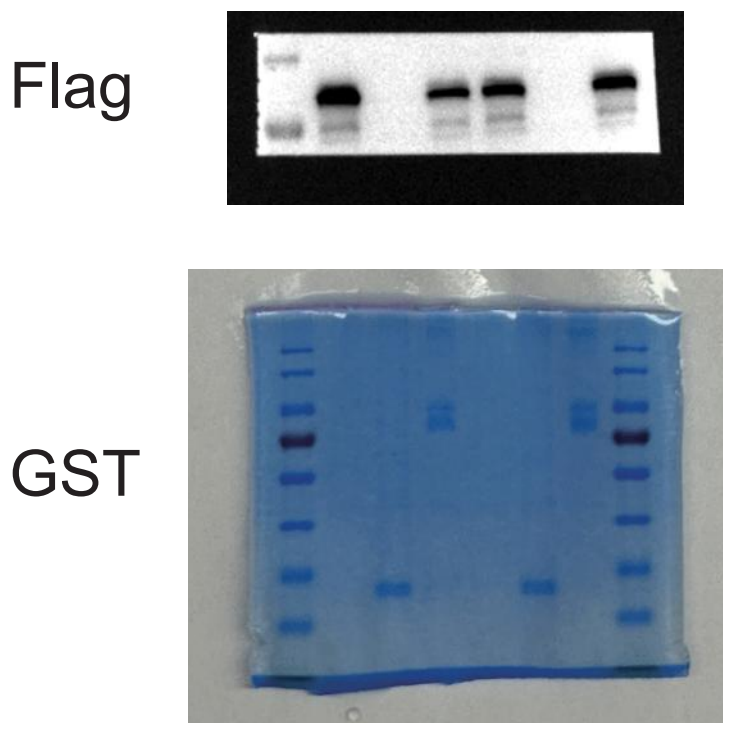

Figure 3I

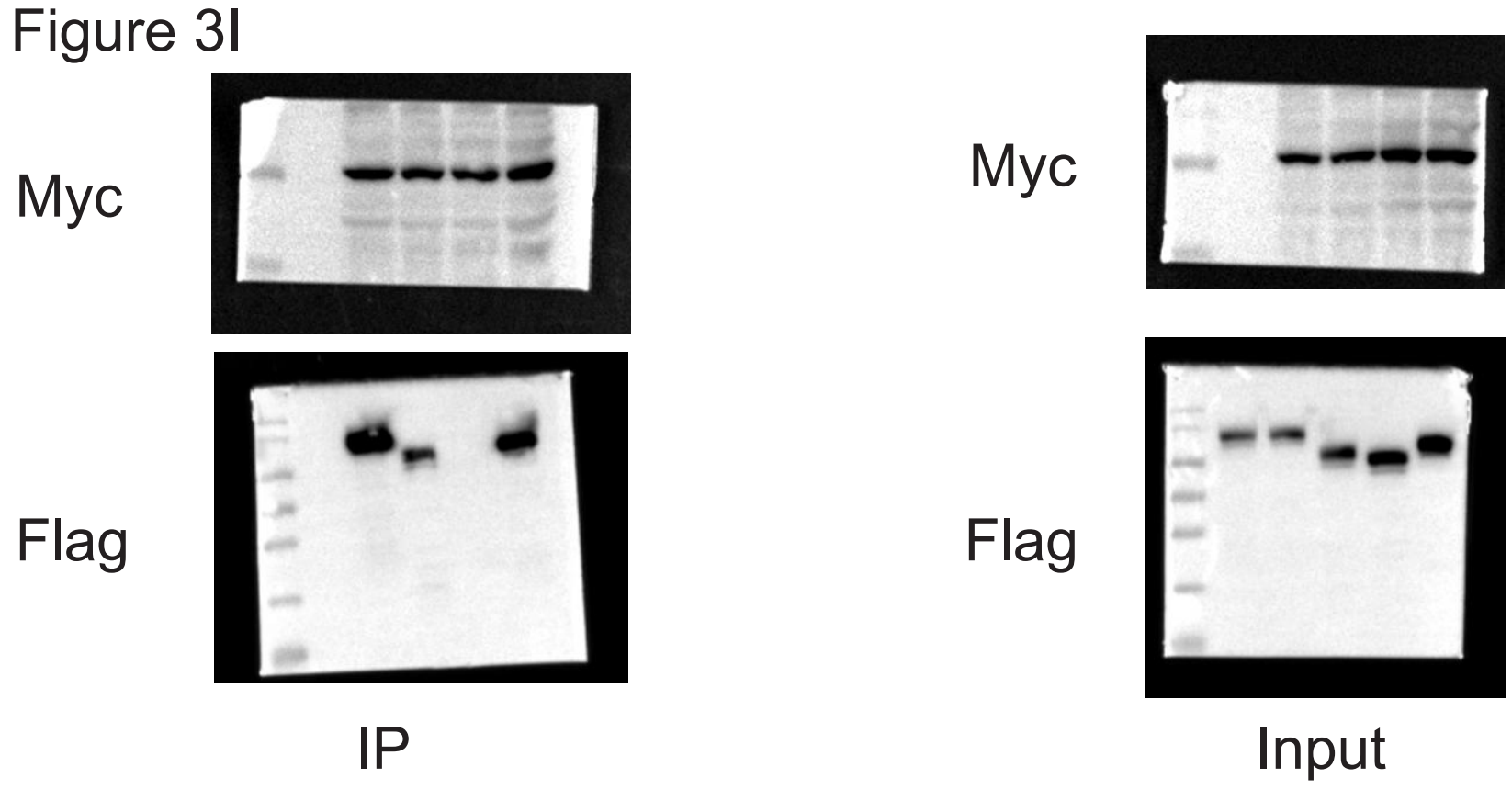

Figure 3J

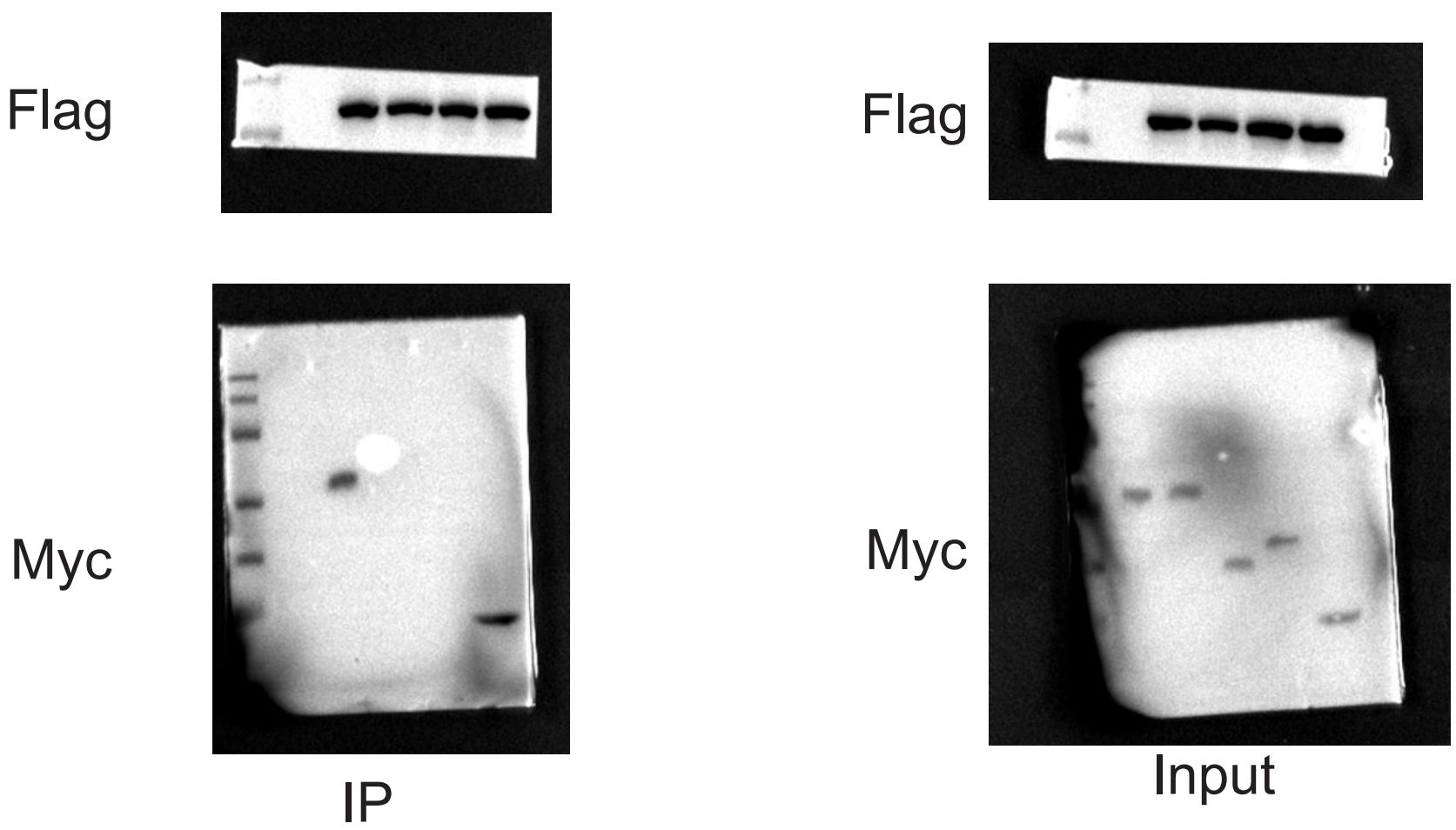

Figure 4A

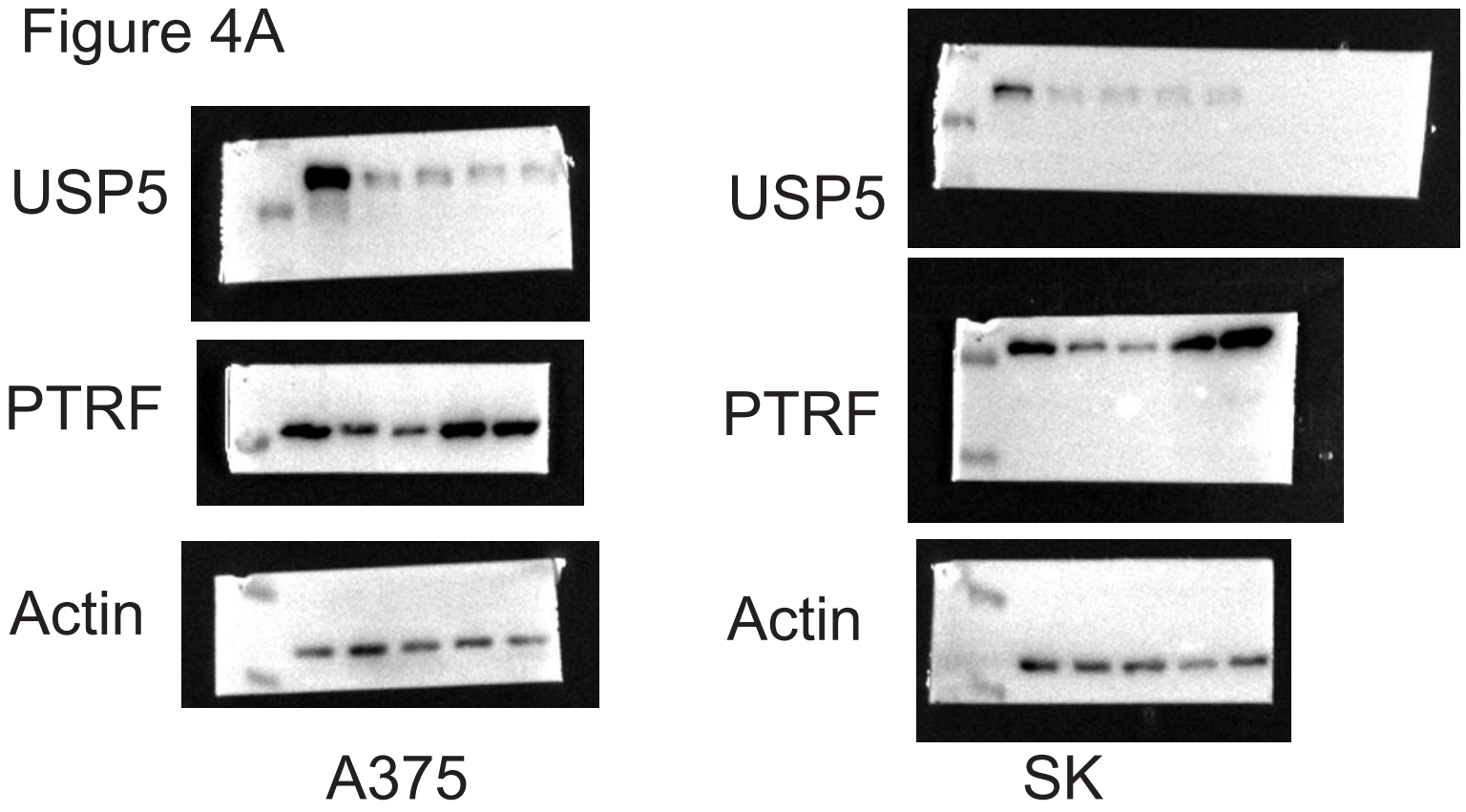

Figure 4B

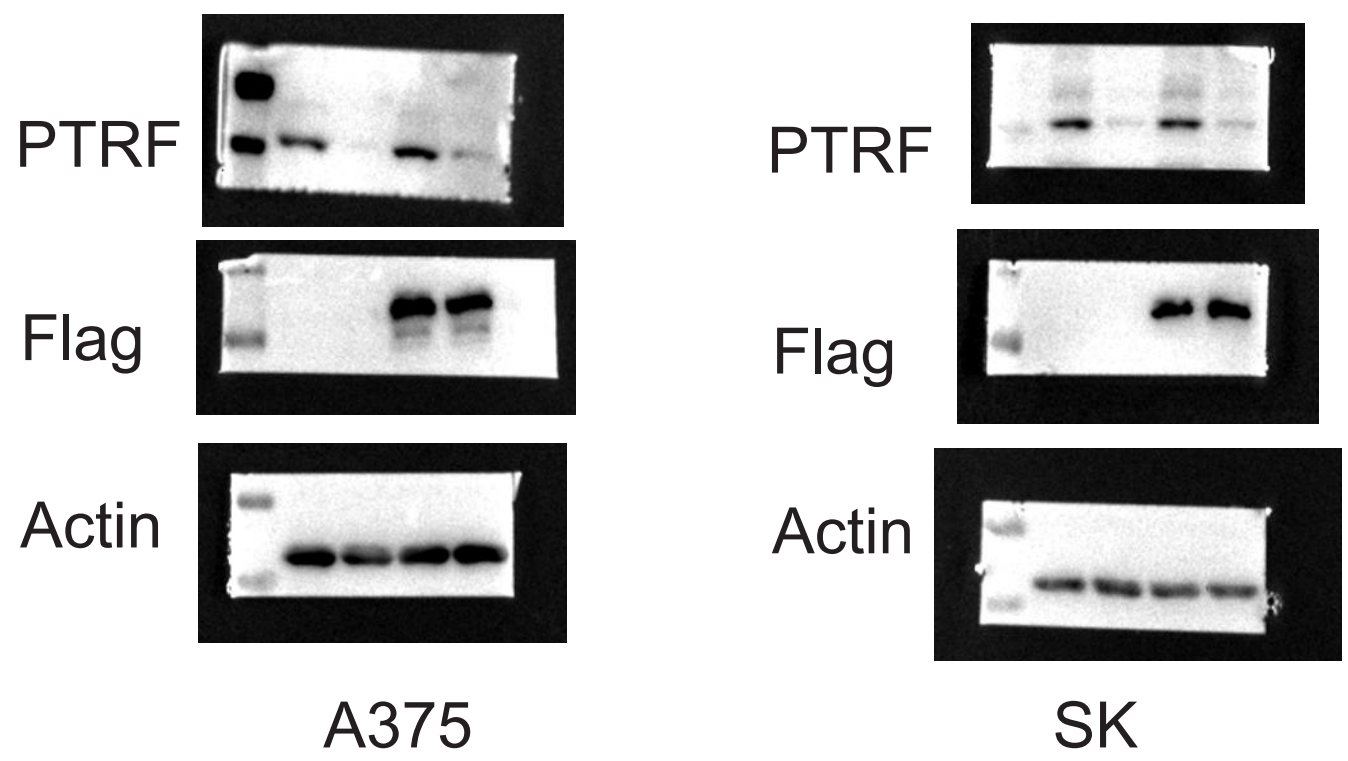

Figure 4D

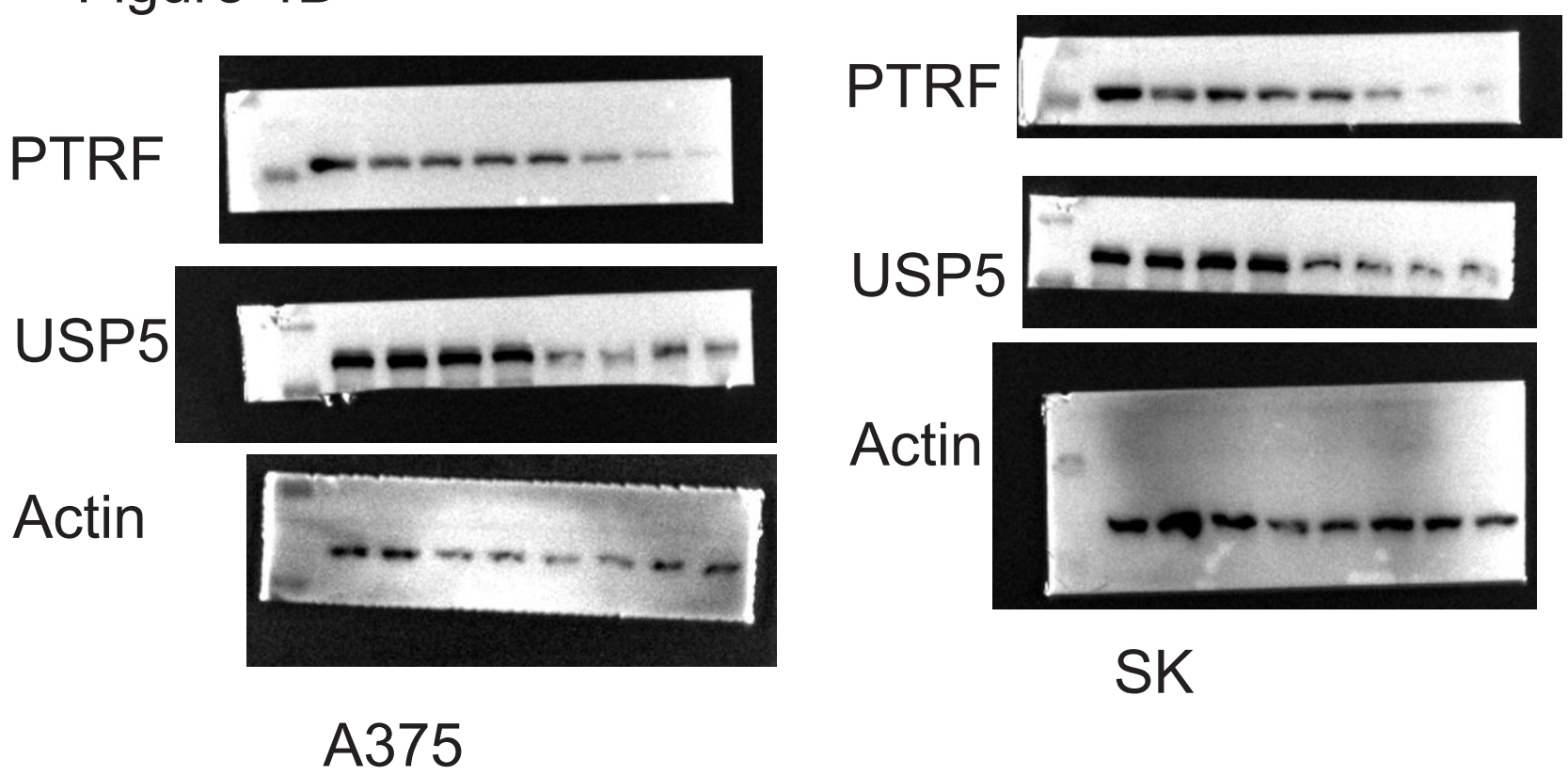

Figure 4F

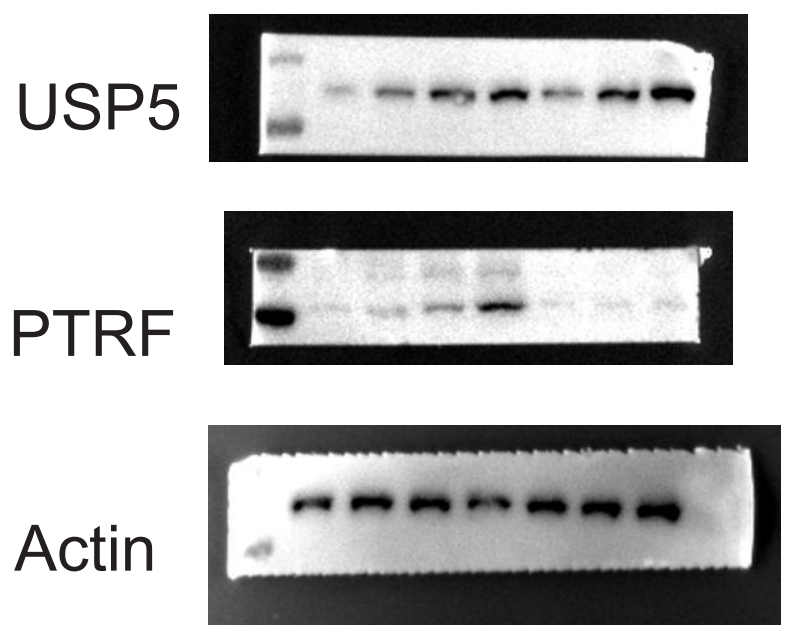

Figure 4G

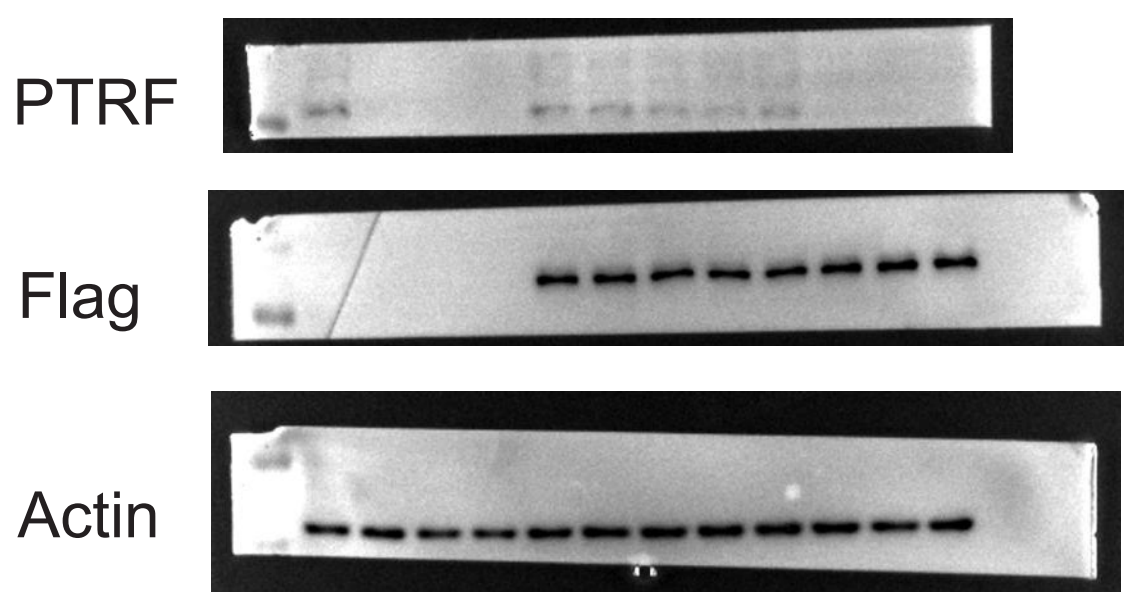

Figure 4I

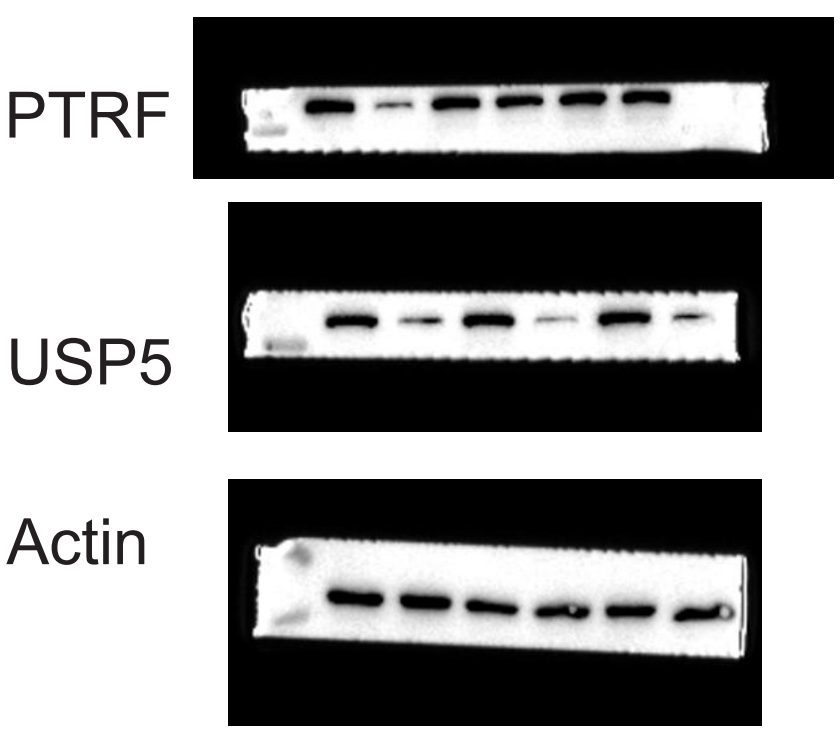

Figure 4J

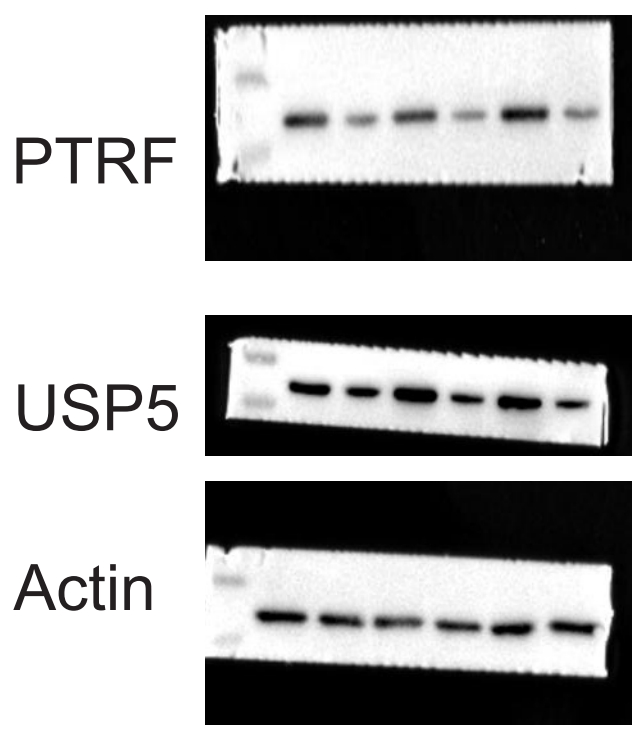

Figure 4K

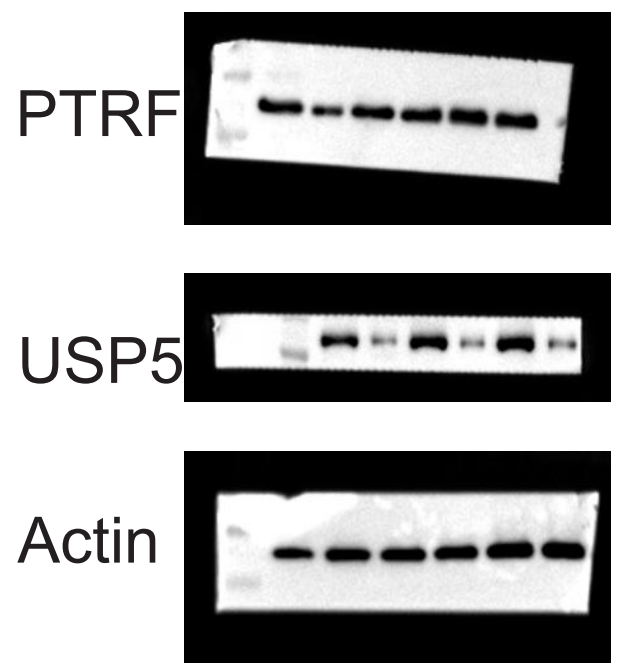

Figure 4L

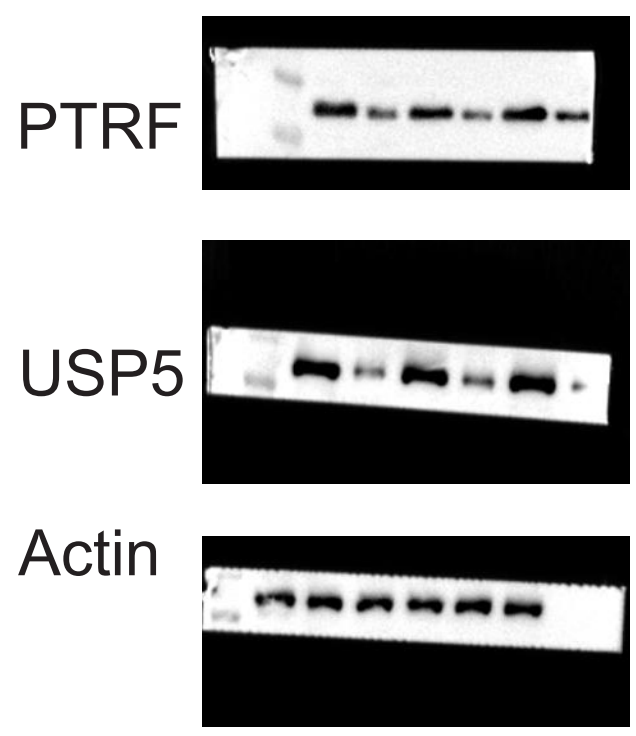

Figure 5A

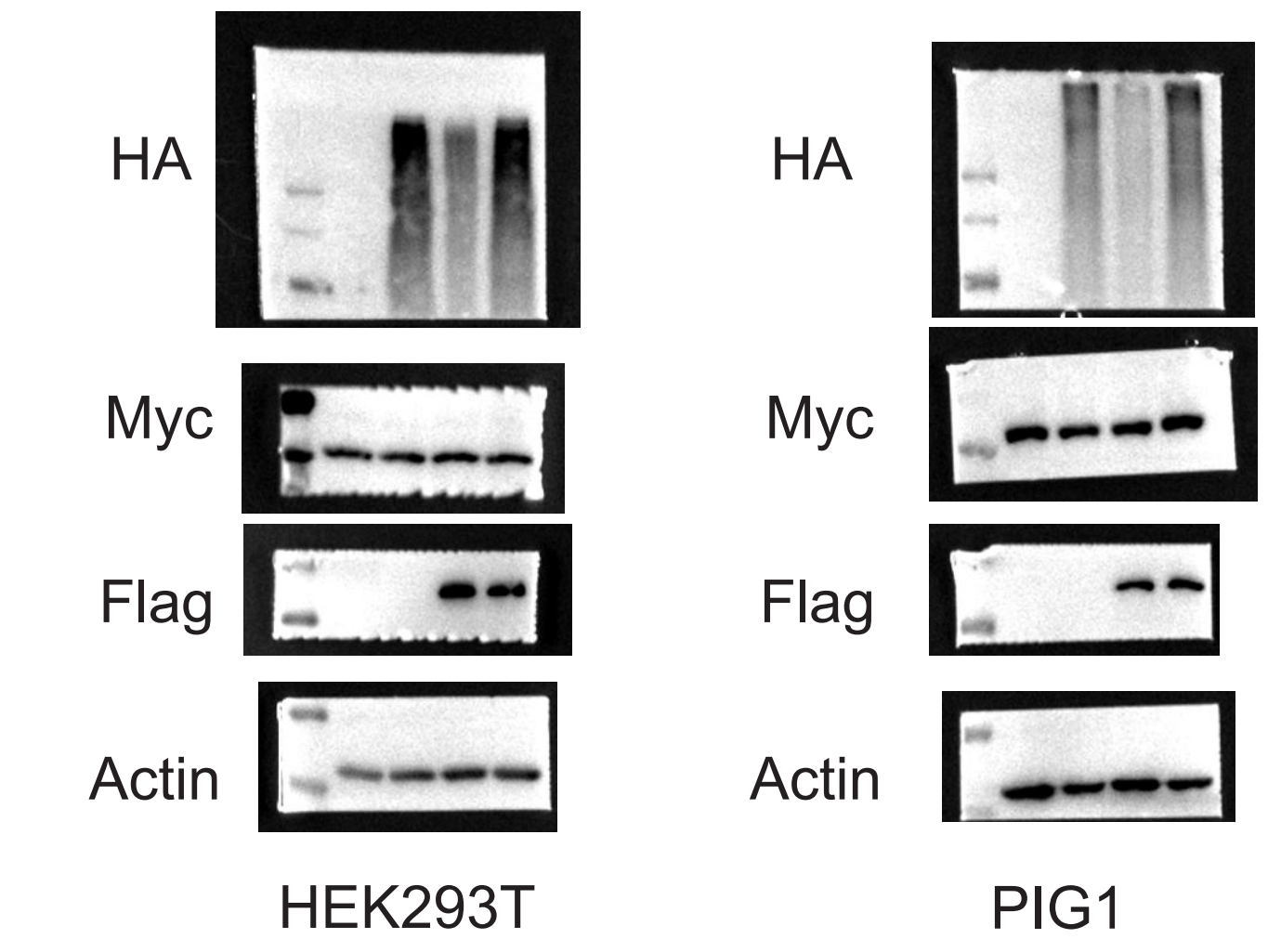

Figure 5B

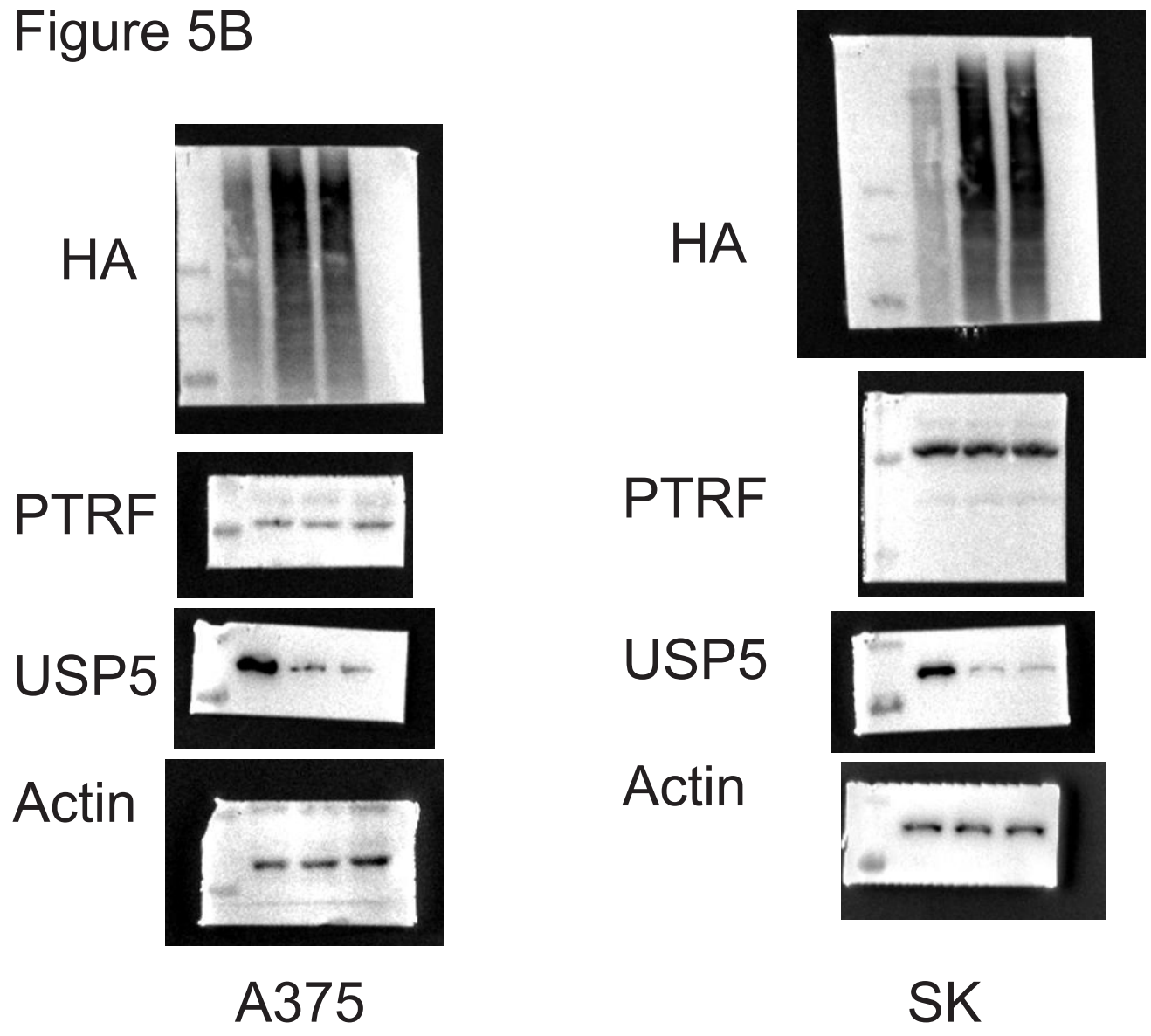

Figure 5C

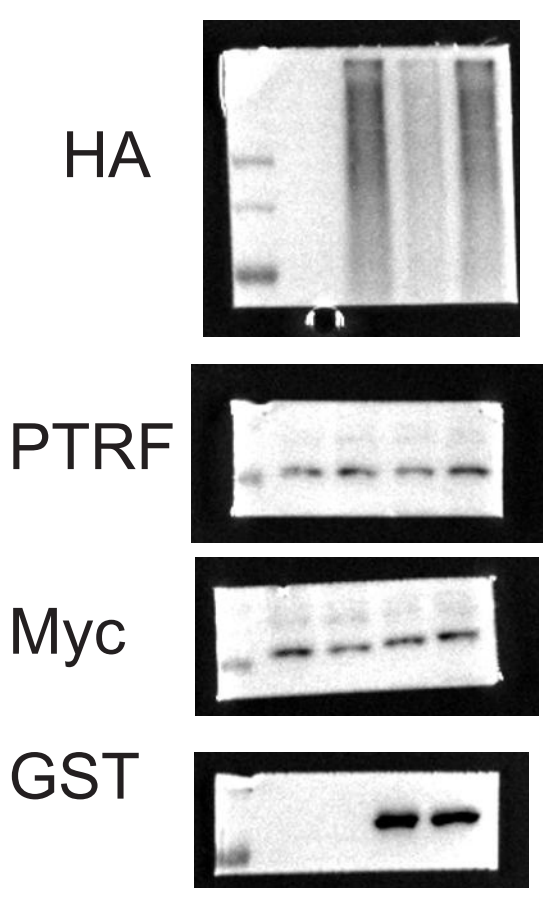

Figure 5D

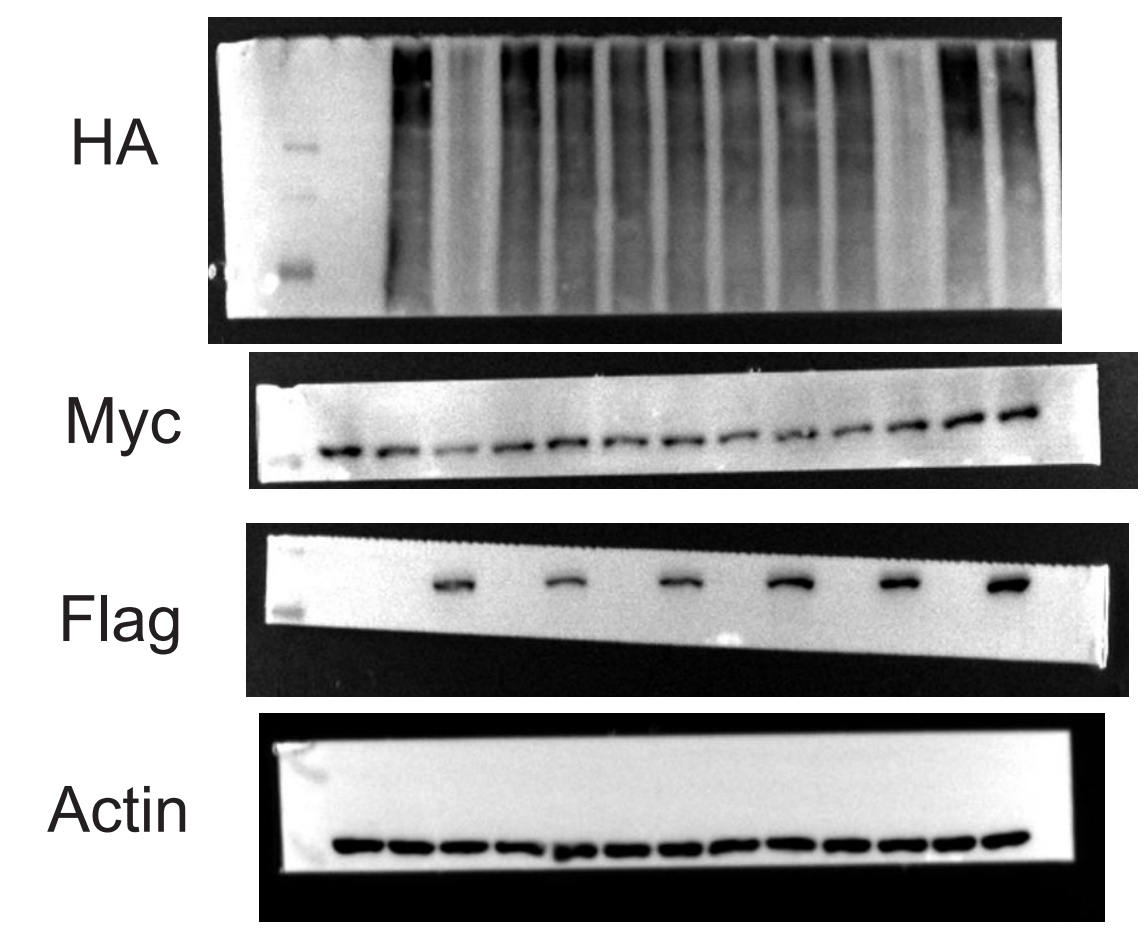

Figure 5E

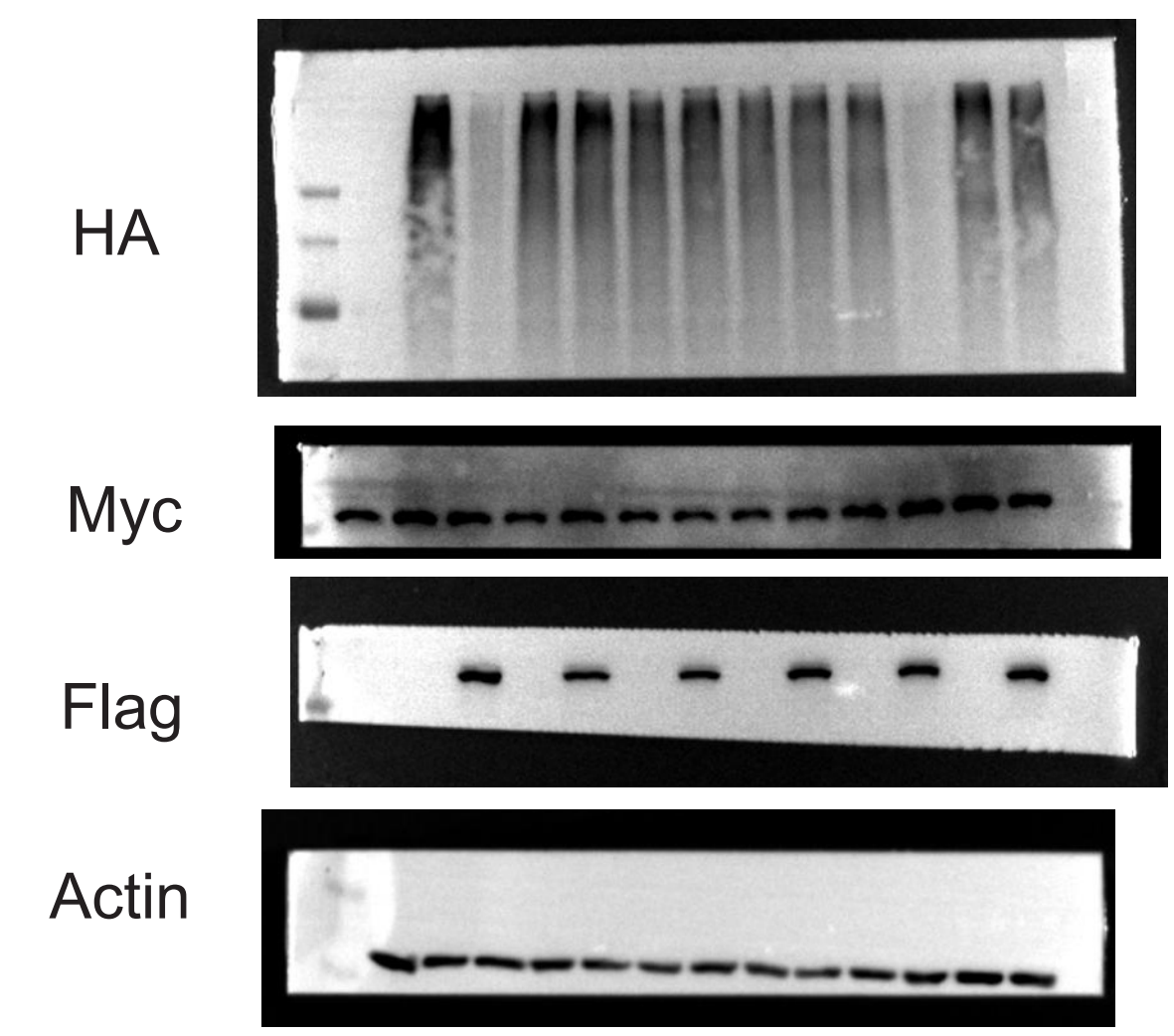

Figure 5F

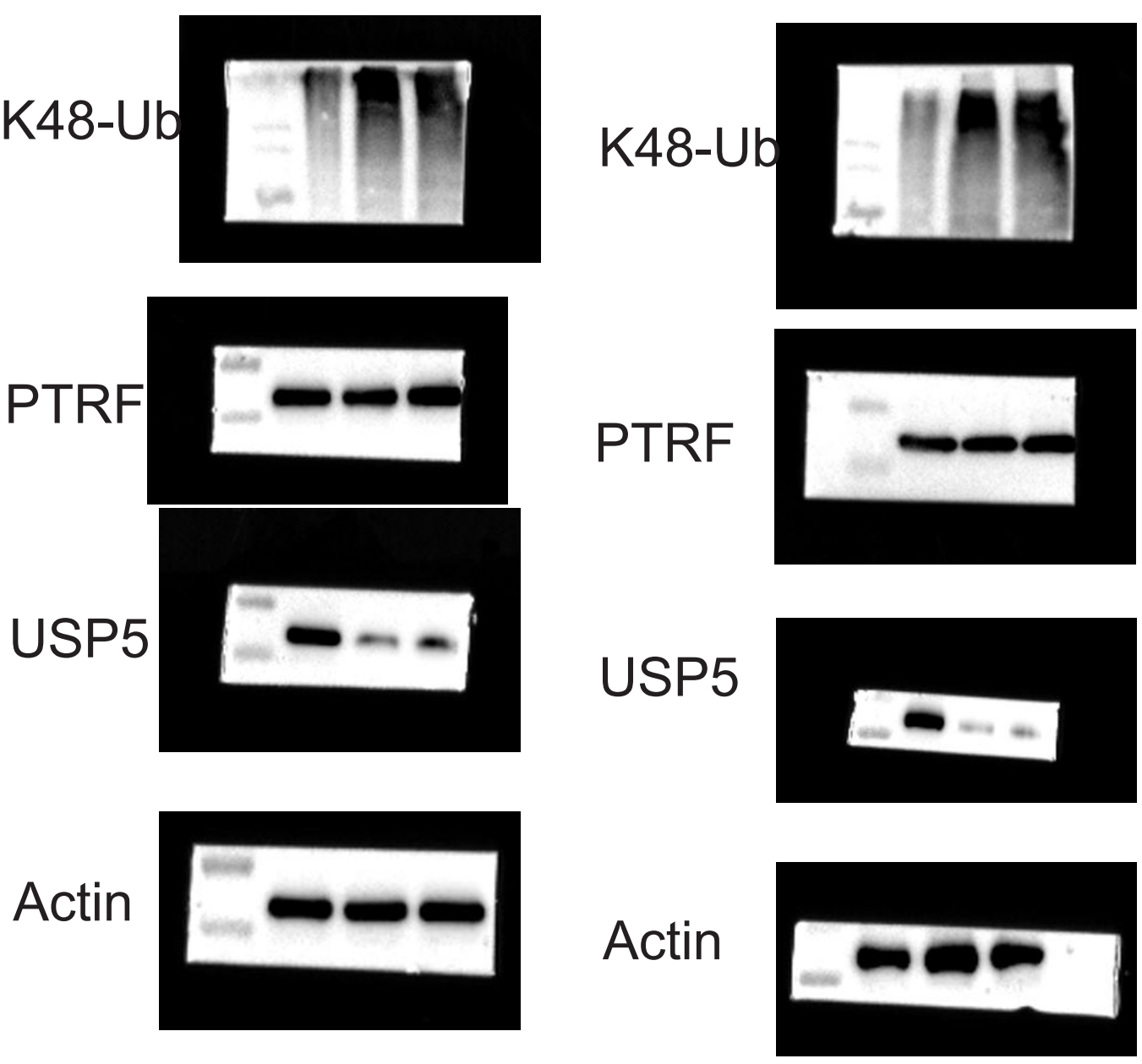

Figure 5G

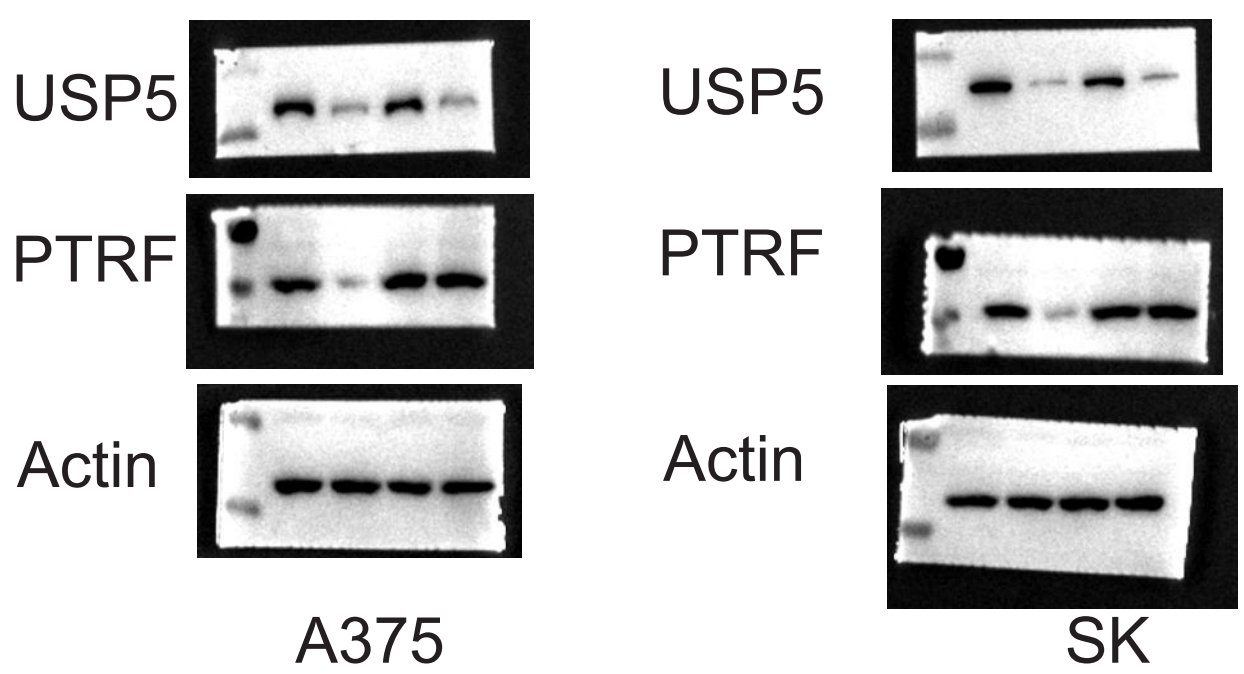

Figure 6A

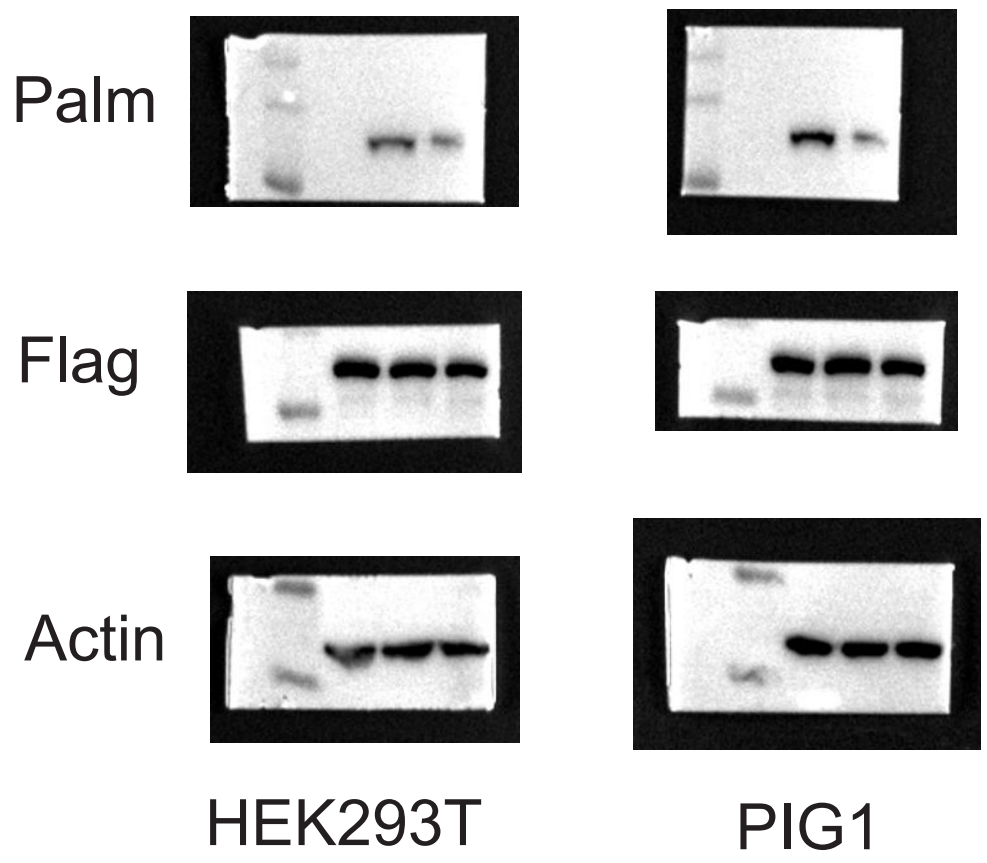

Figure 6B

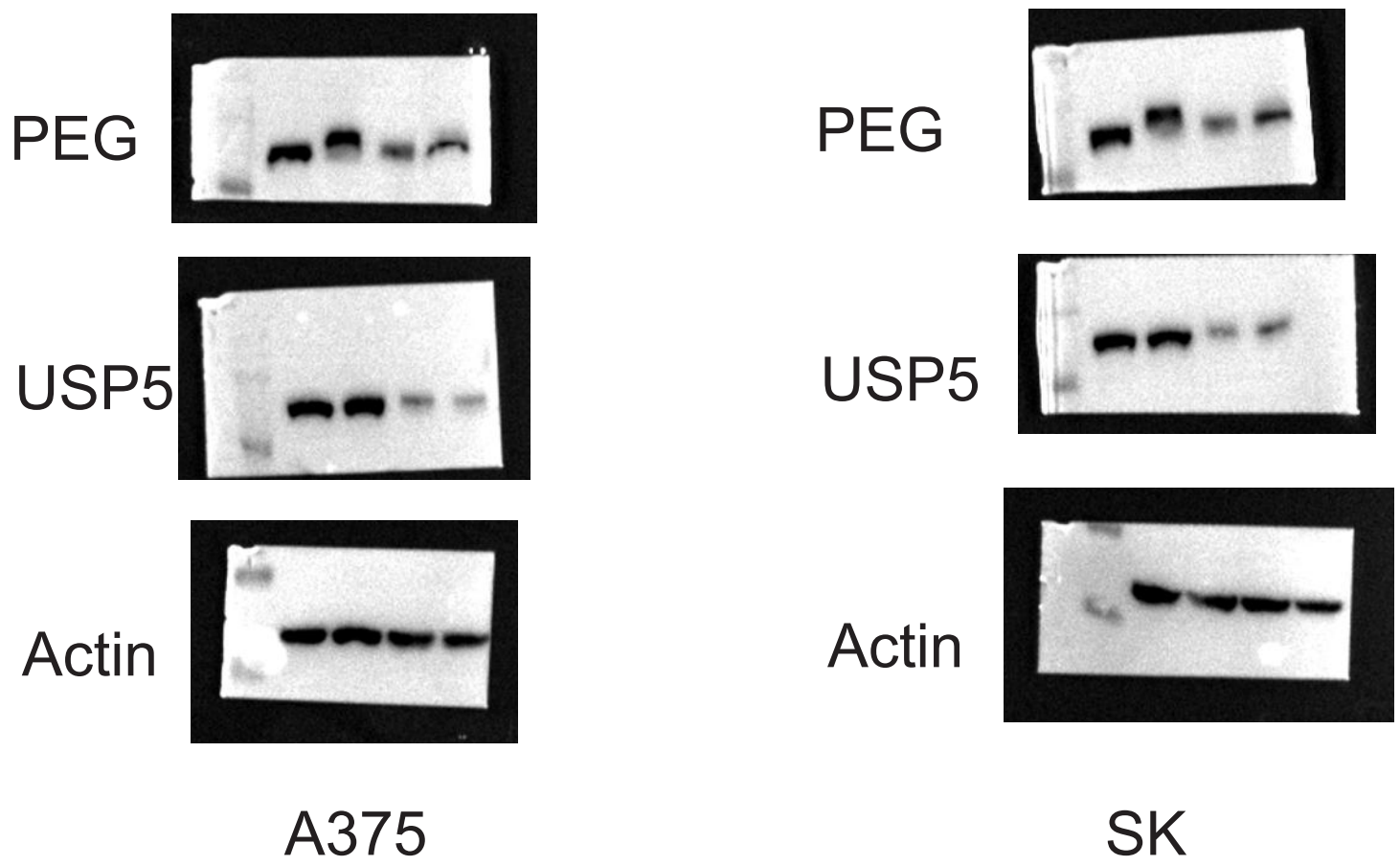

Figure 6C

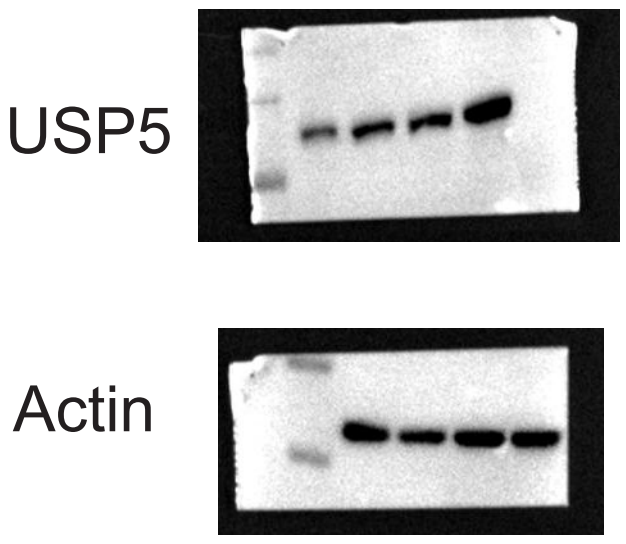

Figure 6D

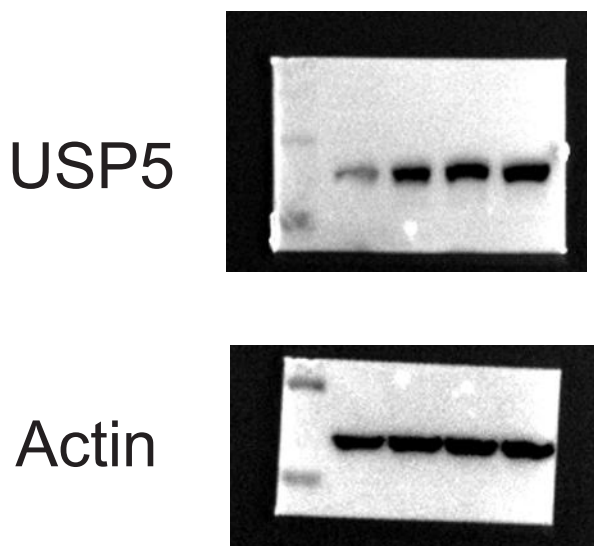

Figure 6E

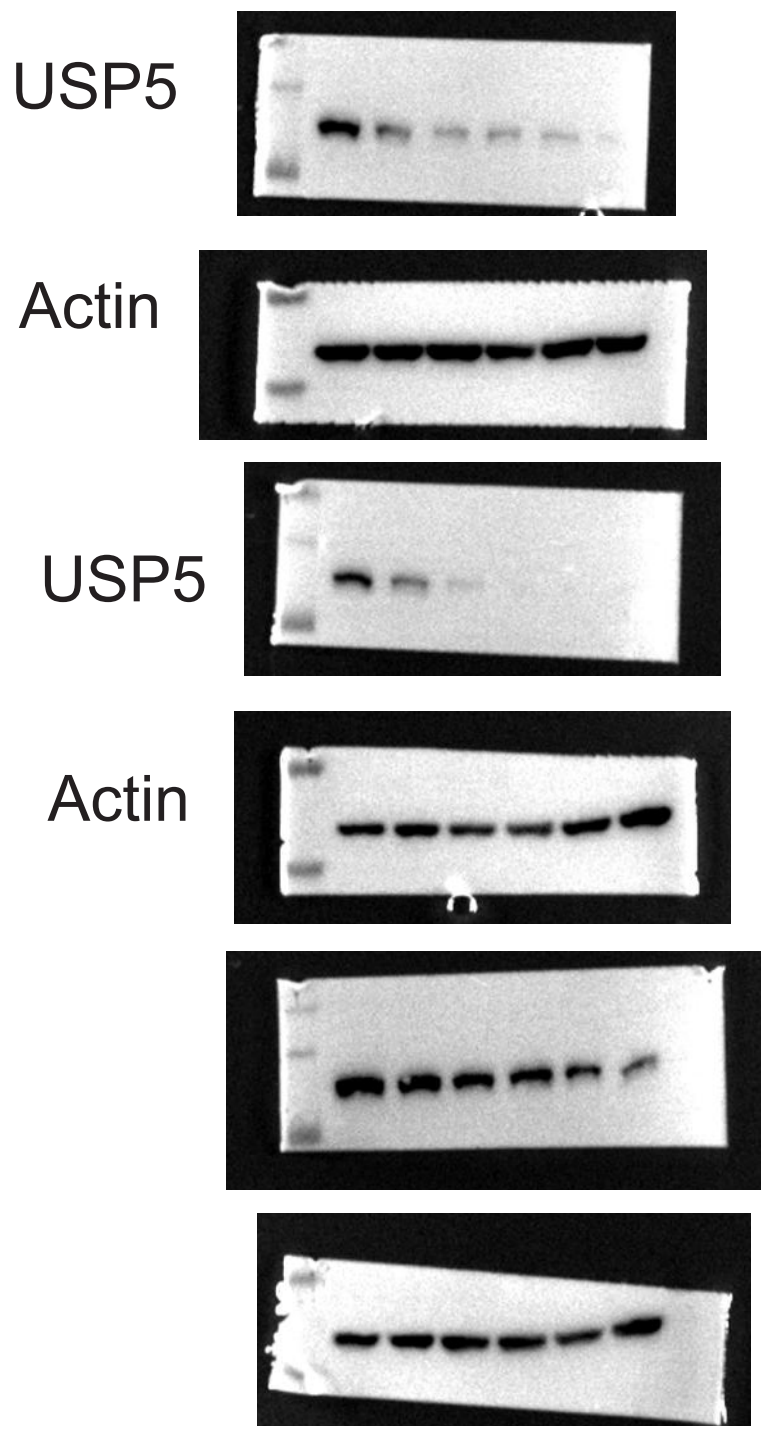

Figure 6F

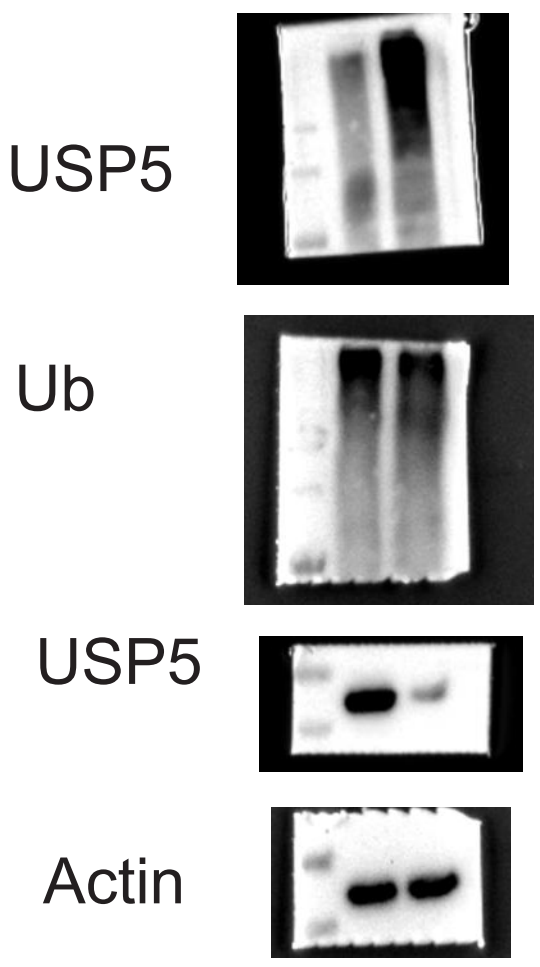

Figure 6G

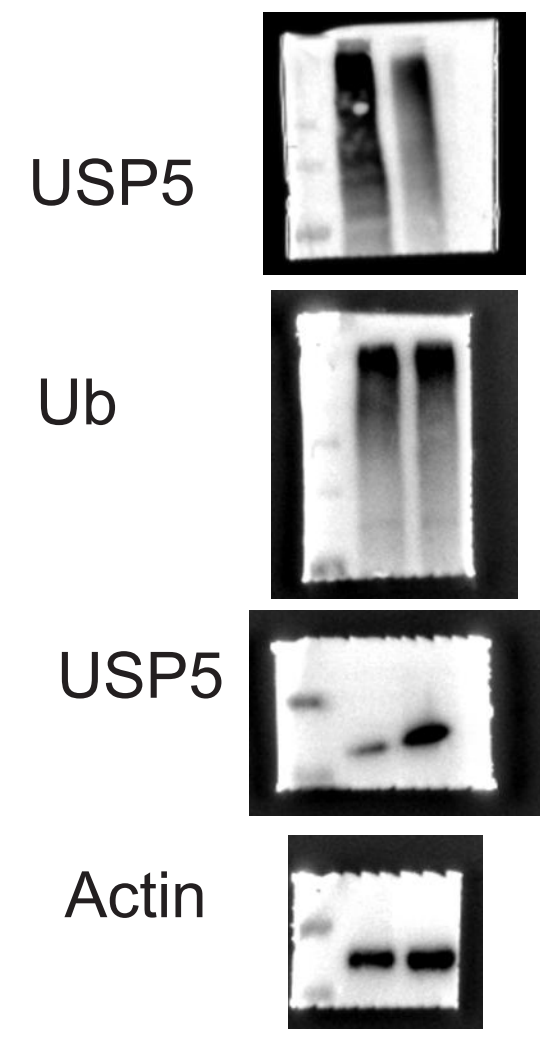

Figure 7B

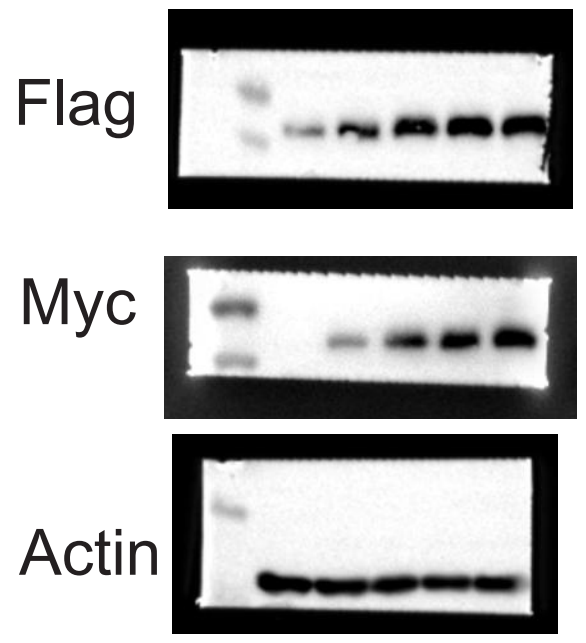

Figure 7C

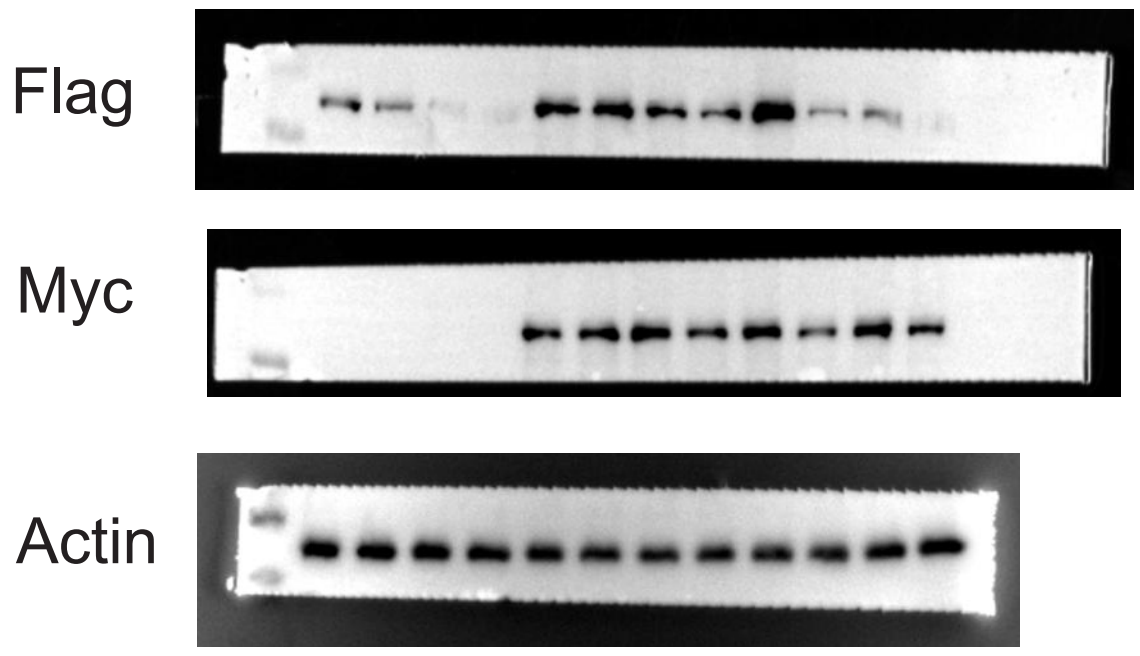

Figure 7D

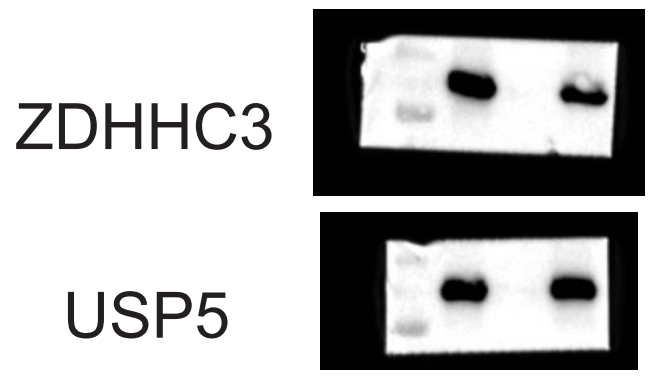

Figure 7E

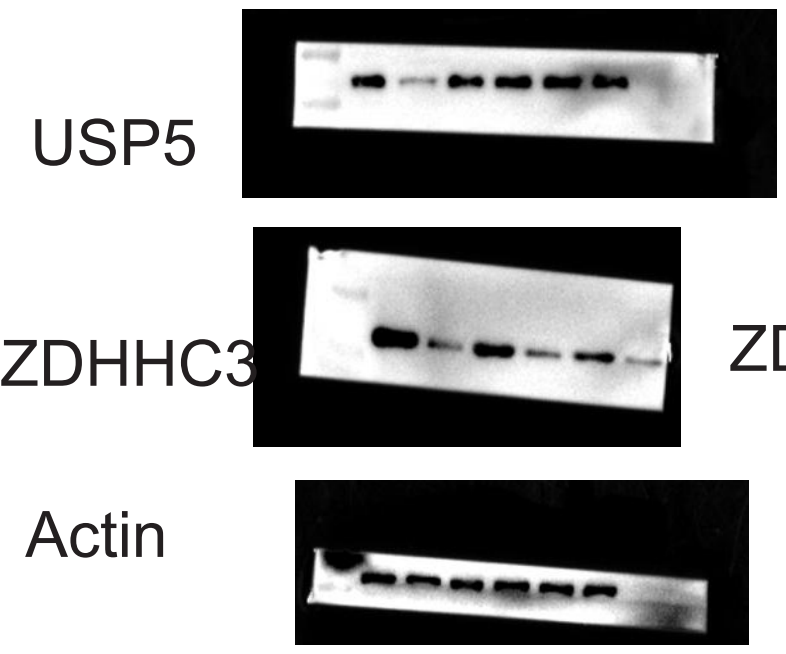

Figure 7F

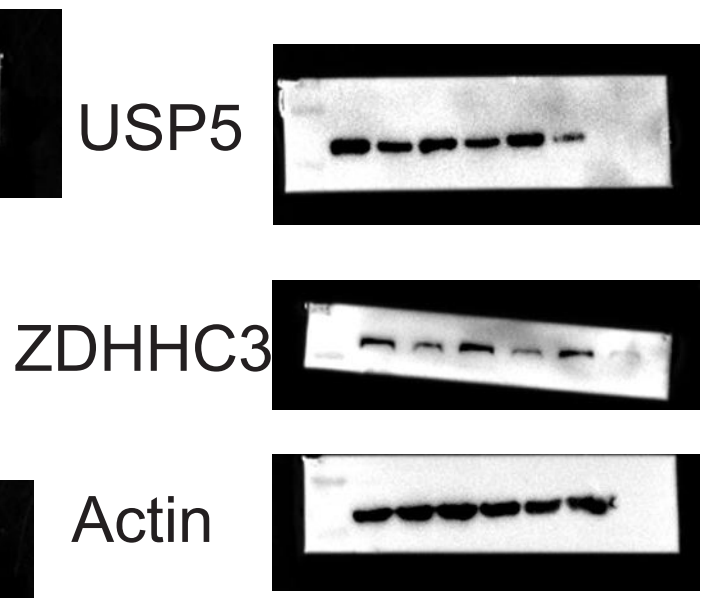

Figure 7G

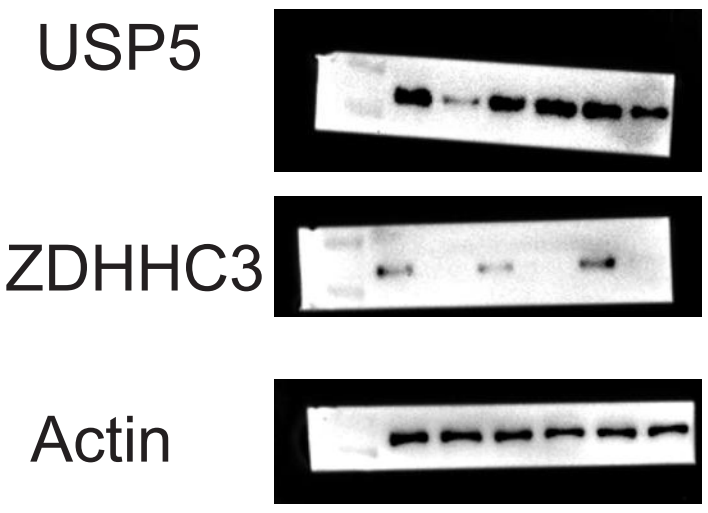

Figure 7H

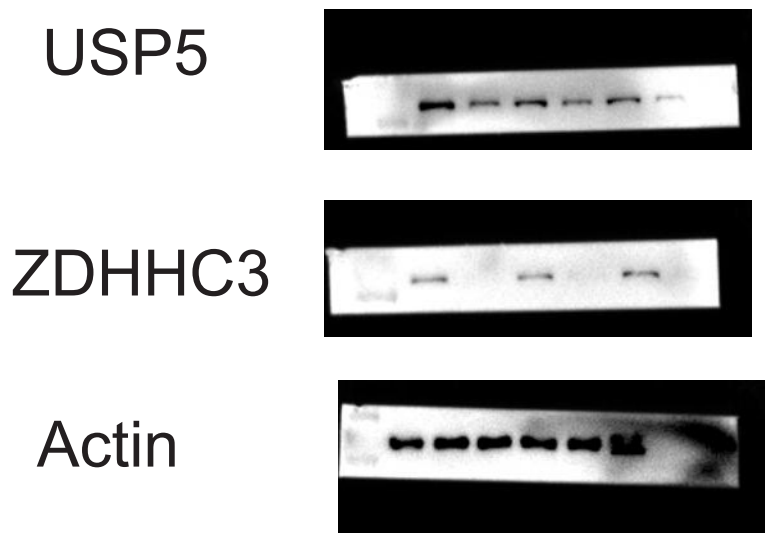

Figure 7I

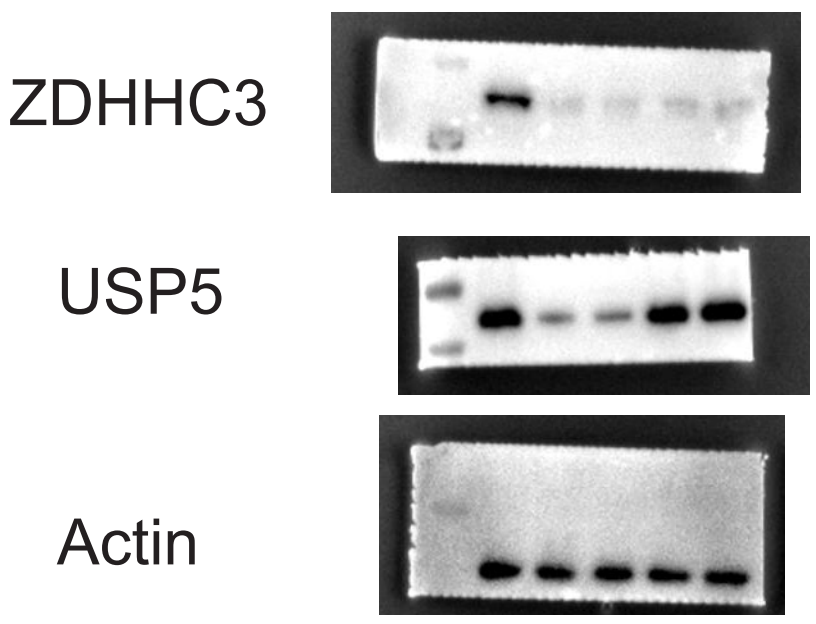

Figure 7J

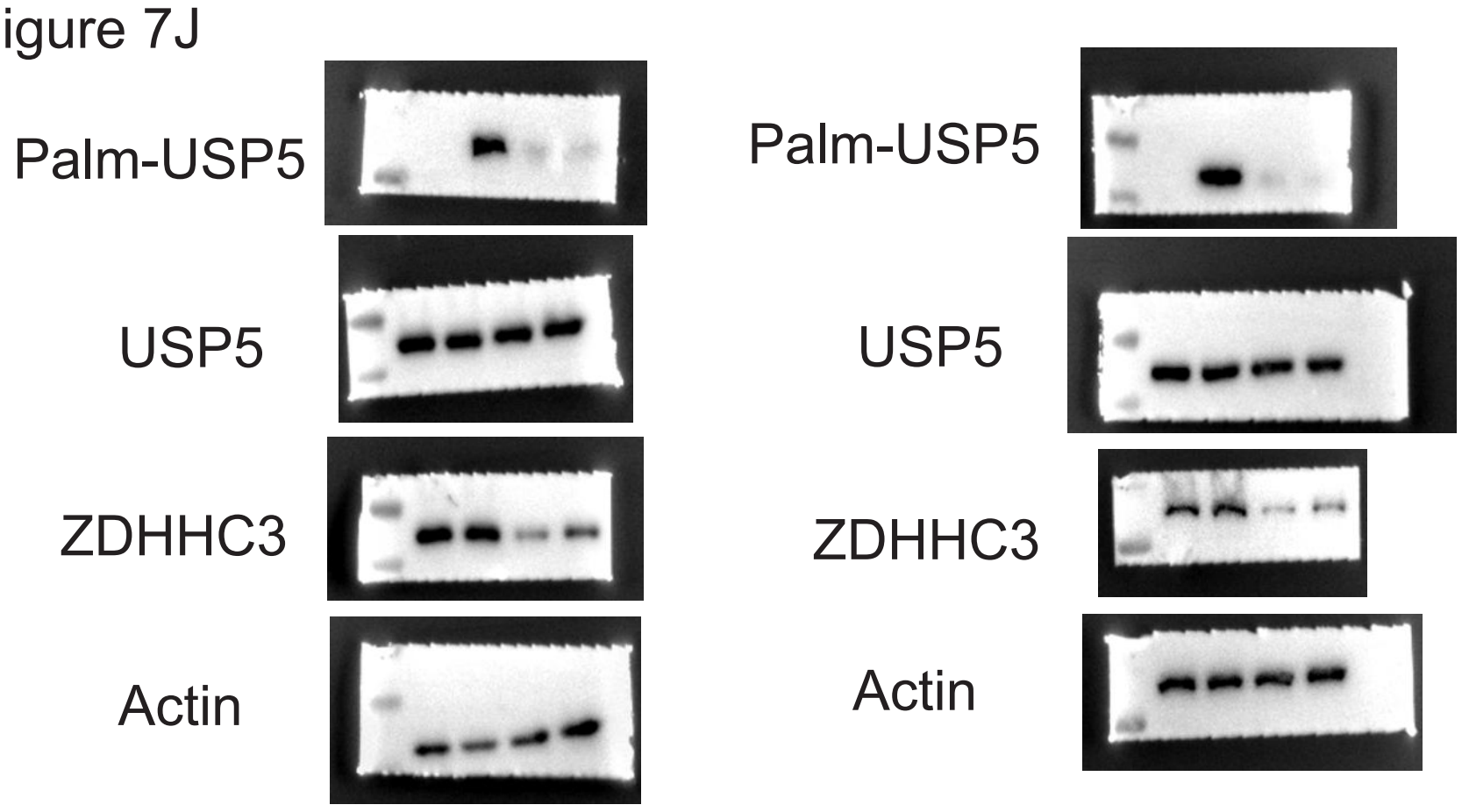

Figure 7K

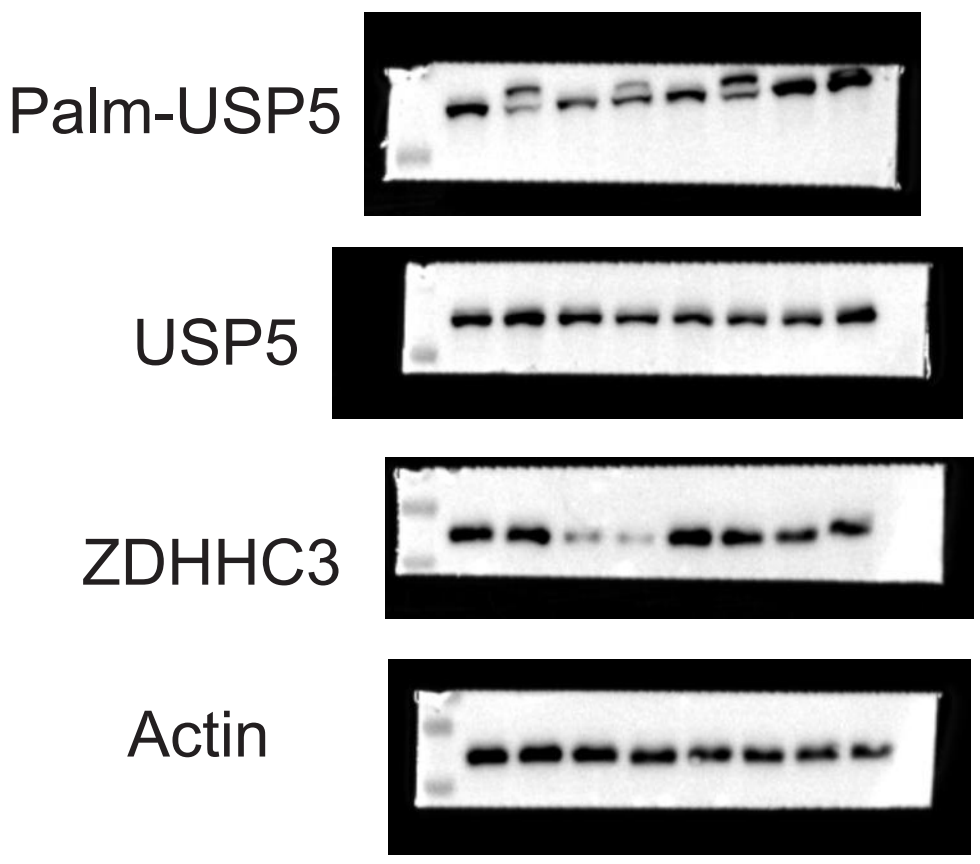

Figure 7L

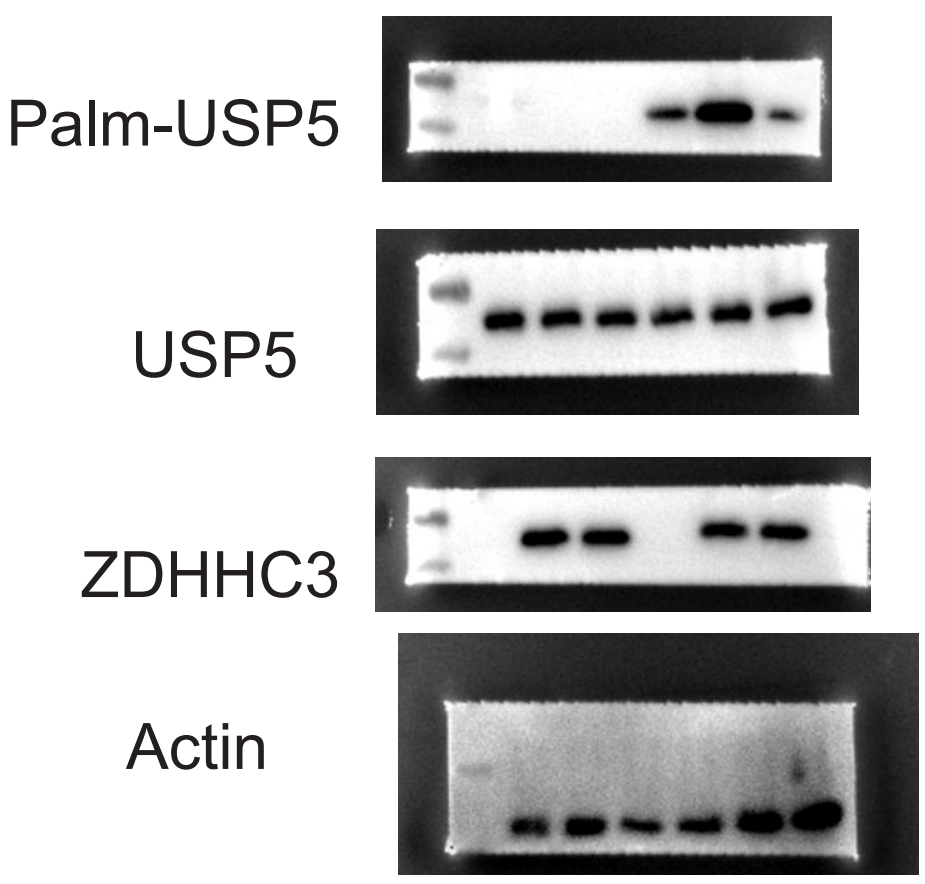

Figure 7M

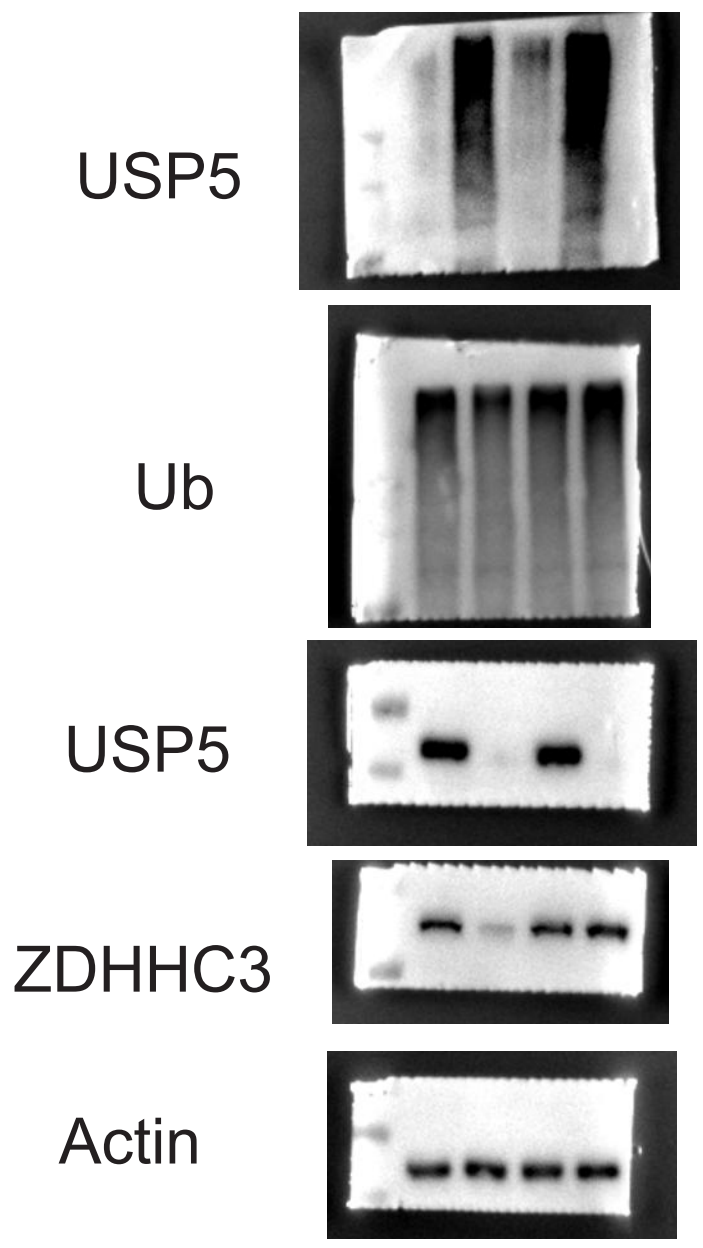

Figure 7N

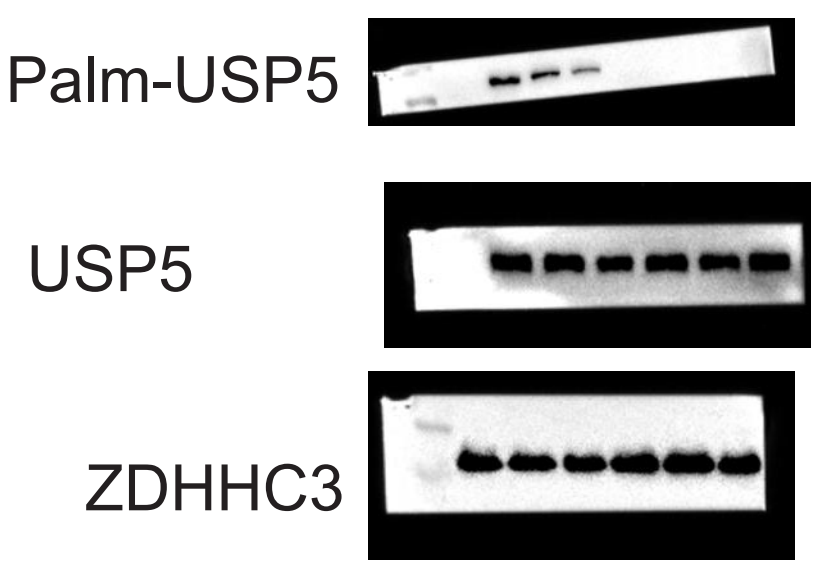

Figure 8A

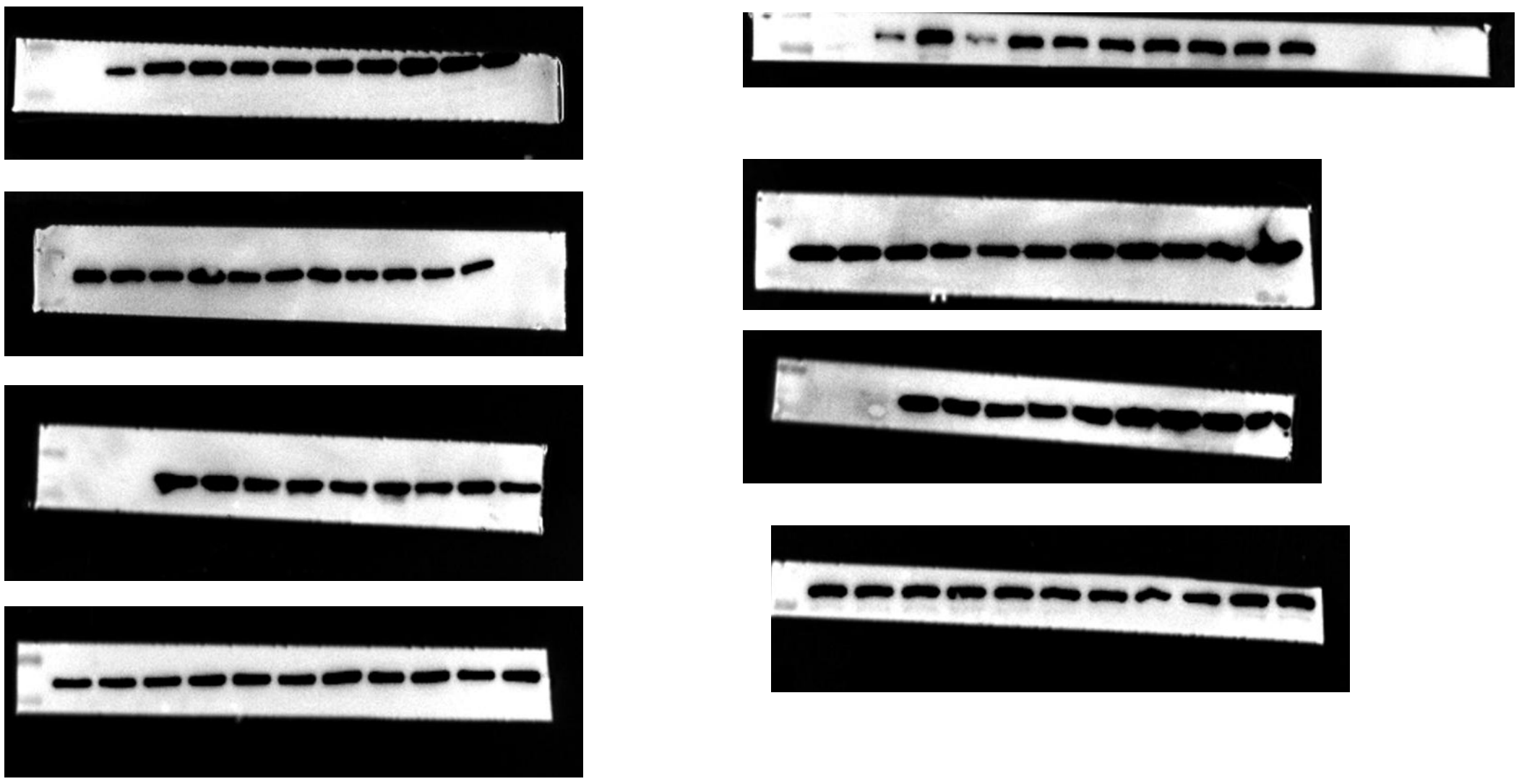

Figure 8B

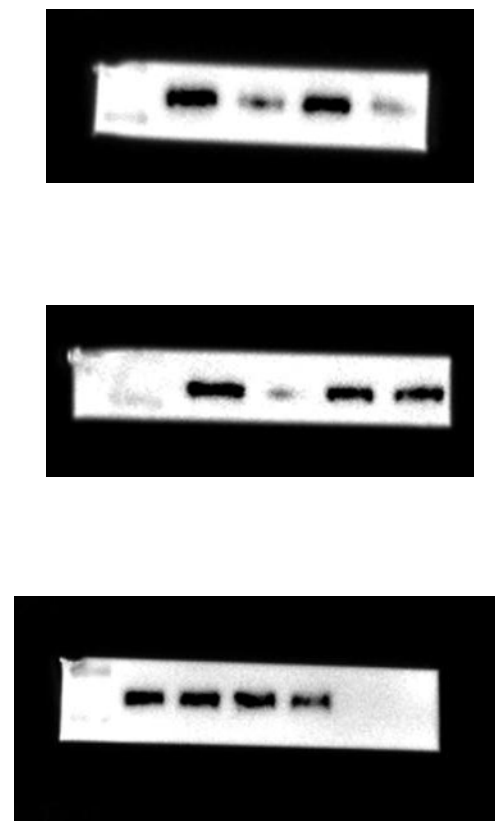

Supplement: Supplementary file 1 [file 41419_2026_9053_MOESM1_ESM.pdf]
